# A new species of *Leptopelis* (Anura, Arthroleptidae) from the south-eastern slope of the Ethiopian Highlands, with notes on the *Leptopelis gramineus* species complex and the revalidation of a previously synonymised species

**DOI:** 10.3897/zookeys.1023.53404

**Published:** 2021-03-11

**Authors:** Arthur Tiutenko, Oleksandr Zinenko

**Affiliations:** 1 Friedrich-Alexander-Universität Erlangen-Nürnberg, Schlossplatz 4, 91054, Erlangen, Germany Friedrich-Alexander-Universität Erlangen-Nürnberg Erlangen Germany; 2 V. N. Karazin National University, 4 Svobody sq., 61058, Kharkiv, Ukraine V. N. Karazin National University Kharkiv Ukraine

**Keywords:** Bale Mountains, Ethiopia, Harenna Forest, *Leptopelis diffidens* sp. nov., *Leptopelis montanus* nom. nov., *Leptopelis rugosus*, *Pseudocassina ocellata*, *Pseudocassina rugosa*

## Abstract

A new ground-dwelling species of treefrog in the genus *Leptopelis* is described from the Harenna Forest in south-eastern Ethiopia. The description is based on morphology and acoustics and is supported by molecular data. The new species has a small body size, and the digital discs on fingers and toes are significantly more conspicuous than in other semi-fossorial members of the *L. gramineus* complex. It occupies forest habitats at lower altitudes and is separated ecologically and geographically from high-altitude species of the complex. One of them, a parapatric cryptic species from Bale and Arsi Mountains, is resurrected from synonymy of *L. gramineus* and given a new name, *L. montanus*. Genetic barcoding of specimens from both populations showed that they belong to two distinct lineages that had been revealed by recent phylogenetic research. To confirm the geographic separation of the studied populations, the collection area of *L. gramineus* types was verified through analysis of the diary and the final report of the 2^nd^ expedition of V. Bottego, and through matching of the route described in it with modern maps. The type locality of *L. gramineus* sensu stricto is restricted to Gamo Gofa, Ethiopia. Following the results of recent phylogenetic studies, the range of *L. gramineus* is limited to west of the Great Rift Valley. An identification key to the named Ethiopian species of the genus is provided.

## Introduction

The genus *Leptopelis* Günther, 1859 is usually referred by the vernacular name ‘Forest Treefrogs’, or just ‘Tree Frogs’. Indeed, the majority of its currently recognised ca. 50 members are arboreal or semi-arboreal frogs, found on tall grass, scrubs, and trees in various habitats across sub-Saharan Africa (Channing and Rödel 2019). A few members of the genus have been known to present an exception, being adapted to semi-fossorial way of life and occupying different habitats and ecological niches than the rest of the congeners. Two of these species, *Leptopelis bocagii* (Günther, 1865) and *L. gramineus* (Boulenger, 1898), have long been assumed to comprise several cryptic taxa (Largen 1977; Schiøtz 1999).

For *L. gramineus*, the evolutionary relationships between populations east and west of the Great Rift Valley (GRV) were outlined in the recent works dealing with phylogeography and evolution of amphibians in the Ethiopian Highlands (Mengistu et al. 2012; Freilich et al. 2016; Reyes-Velasco et al. 2018). The GRV separated *L. gramineus* into eastern and western lineages during the early Pliocene, a period that corresponds to major tectonic processes that shaped the geomorphology of the Rift. The complex and diverse relief and climate at both sides of the GRV were preconditions of further divergence in this group. The high levels of genetic differentiation between amphibian populations were revealed by earlier research on mitochondrial genes (Mengistu et al. 2012; Freilich et al. 2016); however, the appropriateness of separation of cryptic species in *L. gramineus* was doubted because little to no variation between populations was found when a small number of nuclear markers had been used. Reyes-Velasco et al. (2018) re-assessed genome-wide levels of the divergence between species and populations in a more recent study involving both, mtDNA and ddRAD loci. Currently, at least five species are assumed to exist in the *L. gramineus* complex. The same phylogenetic study revealed genetic isolation of the forest population and high-altitude population of *L. gramineus* in the south of the Somali Plateau and confirmed the conclusions that we had drawn from observations we made in 2012–2019 and from material obtained during our fieldwork in the Harenna Forest, one of the last remaining natural forests in this part of Africa.

## Material and methods

### Material examined

The examined and compared material in museum collections comprises 105 specimens identified as *L. gramineus* and 86 specimens of six other *Leptopelis* species from the Horn of Africa. The list of these specimens is provided in Suppl. material 6: Table S2. For collection names we use here the following abbreviations:

**BMNH**Natural History Museum, London, UK;

**LIV**World Museum, National Museums Liverpool, UK;

**MSNG**Museo Civico di Storia Naturale “Giacomo Doria”, Genoa, Italy;

**PEM**Bayworld, Port Elizabeth, South Africa;

**ZMB**Naturhistorisches Museum, Berlin, Germany;

**ZNHM**Zoological Natural History Museum at Addis Ababa University, Addis Ababa, Ethiopia;

**ZSM**Zoologische Staatssammlung, Munich, Germany.

### Morphological measurements and analysis

Morphometric measurements of 45 preserved specimens of three lineages of *L. gramineus* complex were performed with digital callipers with ± 0.1 mm accuracy by the first author. For measured values see Suppl. material 5: Table S1.

The following abbreviations (mainly adopted from Watters et al. 2016) are used for measured parameters: **SVL** = snout-vent length (direct line distance from tip of snout to posterior margin of vent), **HW** = head width (at the widest point), **HL** = head length (from the posterior of the jaws to the tip of the snout), **ED** = eye diameter (horizontally from the anterior to posterior corner of the eye), **EN** = eye-naris distance (from the anterior corner of the eye to the posterior margin of the nostril), **NS** = naris-snout distance (from the centre of a naris to the snout tip), **SL** = snout length (from the tip of the snout to the anterior corner of an eye), **IOD** = interorbital distance (the shortest distance between the orbits), **UEW** = upper eyelid width (greatest width of the upper eyelid margins), **IND** = internarial distance (shortest distance between the inner margins of the nostrils), **TD** = tympanum diameter (greatest horizontal width of the tympanum), **ETD** = eye-tympanum distance (from the anterior margin of the tympanum to the posterior corner of the eye), **FLL** = forearm length (from the flexed elbow to the base of the outer palmar tubercle), **Fin1L** = finger I length (from the proximal edge of the palmar tubercle to the tip of finger I), **Fin2L** = finger II length (from the proximal edge of the palmar tubercle to the tip of finger II), **Fin2W** = finger II terminal phalanx width (measured in the middle of the phalanx), **Fin2DW** = finger III disc width, **Fin3L** = finger III length (from the proximal edge of the palmar tubercle to the tip of finger III), **Fin4L** = finger IV length (from the proximal edge of the palmar tubercle to the tip of finger IV), **Fin4W** = finger IV terminal phalanx width (measured in the middle of the phalanx), **Fin4DW** = finger IV disc width (measured at the widest point of finger IV disc), **TL** = tibia length (distance from the outer surface of the flexed knee to the tibio-tarsal inflection), **THL** = thigh length (distance from the vent to the knee), **TSL** = tarsus length (from the tibio-tarsal articulation to the base of the inner metatarsal tubercle); **Toe1L** = toe I length (measured from the joint of basal phalanx), **Toe2L** = toe II length (measured from the joint of basal phalanx to the toe tip), **Toe3L** = toe III length (measured from the joint of basal phalanx to the toe tip), **Toe4L** = toe IV length (measured from the joint of basal phalanx to the toe tip), **Toe4W** = toe IV terminal phalanx width (measured in the middle of the phalanx), **Toe4DW** = toe IV disc width (measured at the widest point of toe IV disc), **Toe5L** = toe V length (measured from the joint of basal phalanx to the toe tip), **IMT** = inner metatarsal tubercle length, **IMTW** = inner metatarsal tubercle width (at the widest point), **TailL** = tail length (measured from the vent to the tip of the tail).

Statistical analyses were performed with Statistica 8.0. To increase the statistical sample size, we pooled specimens of all ages and sexes in a single sample. To minimise the differences in measurements that are associated with the size of specimens, we divided these values by SVL. Characters with high proportion of missing values in the dataset were excluded. We performed principal component analysis first, to check if any strong signal exists in the study material. Subsequently, a multivariate canonical discriminant analysis was applied in order to assess the morphological distinctiveness and our ability to separate the species on a basis of external characters only. This analysis was done with specimens assigned to three groups according to their geographic origin within the clades determined in recent phylogenetic studies.

The metamorphosis stage of juvenile specimens and larvae we determined according to the table of Gosner (1960).

### Toponymy and geo-referencing

Localities of collection vouchers and of historical records were geo-referenced with Garmin BaseCamp 4.8.8 and QGIS 3.6. Geographic coordinates at our own study sites were taken with Garmin GPSmap 66^st^. For geographic coordinates and elevation of the localities mentioned in the text see Suppl. material 7: Table S3. The names of settlements are spelled according to the database of National Geospatial-Intelligence Agency (geonames.nga.mil). For other toponyms that have no standardised names we used their spelling suggested us by local people (Oromo).

To ensure that the collection localities of our vouchers in the Harenna Forest do not coincide with the type locality and range of *L. gramineus* sensu stricto, we determined the collection area of *L. gramineus* types through analysis of the diary included in the final report of the 2^nd^ expedition of Vittorio Bottego, and through matching of the route described in it with modern maps.

The authors of phylogenetic studies of Ethiopian *Leptopelis* (Mengistu et al. 2012; Freilich et al. 2016; Reyes-Velasco et al. 2018) used a mix of toponyms, such as names of towns, of historical Abyssinian provinces, or of mountain ranges for their clades. Not all of the clades that they recognised coincide or overlap. Also, their number is different in each of these papers. Therefore, we cannot use any of the existing names here and have designed our own simple system of names that derived from historical Abyssinian toponyms ‘Borana’, ‘Shewa’, ‘Sidamo’, ‘Arsi’, and ‘Bale’. In the list of GenBank sequences (provided in Suppl. material 8: Table S4) we matched these names to the names that Mengistu et al. (2012), Freilich et al. (2016) used Reyes-Velasco et al. (2018) used in their publications. This “translation” aid should improve the understanding of the system of clades that we discuss here.

### Molecular methods

We performed genetic barcoding of specimens from populations that we were studying in order to confirm their identity and to connect our results to the published work of other authors. Total genomic DNA was extracted with NeoPrep DNA kit (NeoGene, Kyiv, Ukraine) from muscle tissue samples obtained from *Leptopelis* specimens housed in the Zoological State Collection Munich (ZSM 63/2019, ZSM 80/2019, ZSM 83/2019) and from one non-collected specimen. A fragment of 16S gene was amplified with use of published primers 16Sar and 16Sbr and with reaction conditions as described in Bossuyt and Milinkovitch (2000) and Reyes-Velasco et al. (2018). For this process we used DreamTaq Green PCR Master Mix (2X) (ThermoFisher Scientific). The PCR products were purified according to the ExoSAP protocol and sent to a commercial sequencing company. The chromatograms were visually checked, verified and trimmed. The new sequences are deposited in NCBI GenBank under the following accession numbers: MN909551 (Gaysay Grasslands, Bale Mountains), MN909553 (Harenna Forest), MN909554 (Harenna Forest), MN909555 (Menz-Guassa).

We compared our sequences with already available GenBank records and assigned the populations of our interest to the lineages and haplogroups from previous studies (Mengistu et al. 2012; Freilich et al. 2016; Reyes-Velasco et al. 2018). All similar sequences were retrieved from NCBI GenBank by BLAST search and aligned with MUSCLE algorithm that is implemented in MEGA version X (Kumar et al. 2018). We created a phylogenetic tree with MrBayes 3.2.7a (Ronquist et al. 2012), using the GTR+G substitution model and 1.5 Mio. of generations MCMC, sampling every 100^th^ tree and discarding 10% of trees as burn-in when summarising the results. The parameter convergence was checked using Tracer 1.7.1 (Rambaut et al. 2018). The final tree (see in Suppl. material 11) was visualised and annotated in MEGA version X. Since not all sequences from GenBank had the same length, we performed another analysis using restricted dataset of 50 sequences trimmed to the overlapped region of 435 nucleotides. A phylogenetic network was constructed using statistical parsimony in TCS (Clement et al. 2000). The list of GenBank sequences that we used in this study is provided in Suppl. material 8: Table S4.

### Bioacoustics recordings

We recorded vocalisations of two *Leptopelis* species at 12 localities in the Harenna Forest (Gola, Hacho, Harawa, Haro Alati, Hordoba, Kaffa Guasaa, Manyate, Megano, Segoba, Sire, Woraba, Yagana) and of one parapatric species at one locality in Bale Mountains (Gaysay Grasslands). The taxonomic identity of these populations, hence of calling individuals, had been established through aforementioned genetic barcoding. The sound recordings were performed in May and June 2019 with a TASCAM DR-100MKII stereo recorder, processed with Adobe Audition CC 2019, analysed and visualised with Sonic Visualiser 4.0.1 (University of London). In the Harenna Forest the advertisement calls were recorded in the morning (9–11 a.m.), in the afternoon (2–4 p.m.) and after sunset (9–11 p.m.), at air temperatures of 19–26 °C and at no wind. At the high-altitude locality (Gaysay Grasslands) our activities in dark hours were not allowed for security reasons. We recorded there in the morning and in the afternoon at similar temperatures, however, at wind speed of ca. 30 km/h.

## Results and discussion

Initially we became aware of a possibly new species of *Leptopelis* when we heard advertisement calls during our first trip and stays on glades in the Harenna Forest in March 2012: They differed from vocalisations that we had heard previously in a high-altitude population of *L. gramineus* in the Web Valley, Bale Mountains. We made the same observations during our next trips and recorded the calls in May–June 2019. Comparative examination of specimens from the Harenna Forest and from other populations distributed across Ethiopian Highlands increased our confidence that two species of burrowing *Leptopelis* live in parapatry north and south of the Harenna Escarpment and occupy different habitats. Genetic barcoding of individuals from both populations showed that they belong to two distinct lineages that had been revealed by recent phylogenetic research (Freilich et al. 2016; Reyes-Velasco et al. 2018).

Any further analyses and taxonomic decisions require clarity about the geographic range of *L. gramineus* sensu stricto. Since no species in this complex are sympatric, one of them with the range that includes the type locality can be considered as ‘true’ *L. gramineus*. Like many species of African herpetofauna described in the 19^th^ and early 20^th^ century, the type locality of *L. gramineus* was imprecisely given between two places separated from each other by several mountain ranges and by 180 km straight line. Fortunately, we know the exact route of Bottego’s expedition and can approximately determine the position of collection sites along it, thus restrict the type locality to a more concrete geographic area.

### Restriction of the type locality of *L. gramineus*

The often-used English vernacular name ‘Badditu Forest Treefrog’ seems to have been coined by Frank and Ramus (1995). It is misleading because the type specimens originate from the area “between Badditu and Dime”, according to original description by Boulenger (1898), as well as to BMNH and to MSNG catalogues. Vannutelli and Citerni (1899: 606) who had accompanied Bottego in his expedition wrote in the final report clearly that the frogs, described by Boulenger as two new species, were obtained on the way from Badditu to Dime: “*I due nuovi graziosi Batraci*, *Megalixalus gramineus Blgr. e Hylambates Vannutellii Blgr.*, *furono ambedue raccolti durante il percorso dai Badditù a Dimè.*”

**Figure 1.**
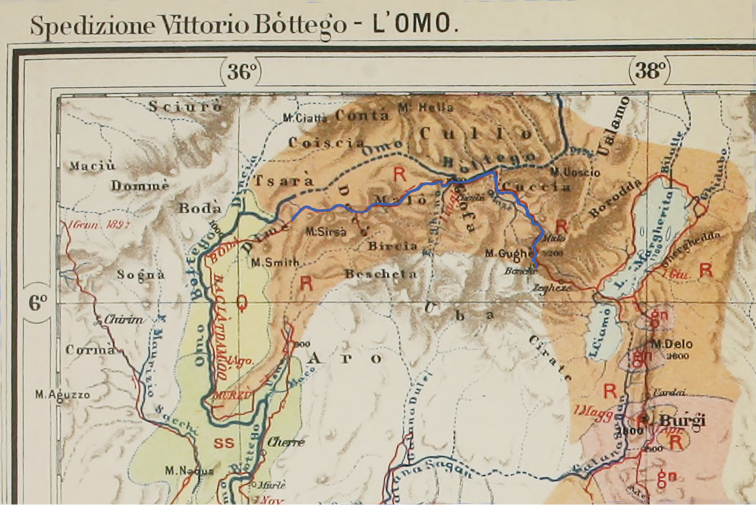
Fragment of a map from Vannutelli and Citerni 1899 showing the route that the 2^nd^ expedition of Vittorio Bottego took in 1896 (dark red line). The part of the route where type specimens of *Leptopelis gramineus* were collected is marked with blue colour.

Since no specimens tagged only with the locality ‘Badditu’ exist, it should be safe for us to assume that *L. gramineus* was not found by Bottego’s expedition at the eastern shores of the lakes Abaya and Chamo, i.e., where the Badditu tribal territory is situated. This place cannot be found on modern maps because it is a historical tribe name that was used as toponym mainly by Italians. The members of this tribe called themselves ‘Koyra’ (Bryan and Tucker 1930; Cerulli 1956), and today this ethnic group is better known as ‘Koora’. Their territory is restricted to an area north of the Burji tribal territory (Fig. 1), on a rugged mountain spur east of Lake Abaya (Cerulli 1956).

Bottego and his people came to Burji on 17 March 1896 (see Fig. 1) and from there went northward, along the eastern side of the GRV, through Badditu territory. They discovered the ‘Lake Margherita’ (now Lake Abaya) on 12 May having already left Badditu. They spent a month exploring this lake that is located in the Rift at elevation of 1175 m a.s.l. and left the area by the middle of June 1896. As for *L. gramineus*, we know that it does not occur at such low elevations, hence the collecting locality could not be there. Moreover, much of the area at the Lakes Abaya and Chamo is today in the popular Necisar National Park. This protected area is well studied, and extensive collections were deposited in a number of museums. *L. gramineus* have never been recorded there.

In the second half of June the Bottego expedition went further west, to explore the lower Omo River, and traversed mountain ranges of over 3000 m in altitude. Finally, they reached Dime – another tribal area around Mount Smith, today situated in northern part of Selamago district in Southern Nation, Nationalities and Peoples Region. After that they never went back to Badditu: In October 1896 the expedition returned to the north corner of Lake Rudolf (now Turkana) and went from there again north where Bottego was killed in a battle in Daga Roba on 17 March 1897.

In his revision of Ethiopian *Leptopelis*Largen (1977) gave an erroneous collection date of the type series, “5 July 1896”, having mistaken May (the 5^th^ month) for the 5^th^ day of July. In his later publication (Largen 2001) he corrected this mistake and used roman numerals for both, May and July: “V–VII.1896”. This agrees with the specimen tags and the catalogue information: Boulenger himself made an entry in the hand-written catalogue of the Natural History Museum that the two specimens he had received (and which are now treated as paralectotypes) had been collected “between” May and July 1896. This also corresponds to what we see on the original specimen tag that is still kept in the glass with the specimens, a handwritten note in Italian (probably by Vannutelli): “*Dai Badditù a Dimè. Magg.–Lugh. 96. Sped. Bottego*”. We do not know for sure whether all specimens of the type series were collected at one single site. However, since not just one locality and date but a time span and a range between two places (Badditu and Dime) are provided, it appears more logical that there were several collecting sites. Obviously, we can exclude Mount Smith, because it is in Dime, and the specimens are known to have been collected on the way there. The Bottego expedition should have crossed approximately four mountain ranges in this area, including Gughe which is in vicinity of what is today Arba Minch town. There are a few specimens of *L. gramineus* from this area in collections, including nearby places Chencha, Dorse, Bonche Valley. The type specimens might have been collected there or on further mountain ranges which offer suitable climatic conditions and habitats (Coltorti et al. 2019). Unfortunately, when Capocaccia (1957) designated the lectotype of *L. gramineus*, she did not validate and restrict the type locality, as recommended by the ICZN, Recommendation 74E. Therefore, the whole area between Mt. Gughe and Mt. Smith, as well as Mt. Gughe itself can still be viewed as such. This Ethiopian province was called Gamu Gofa, and the tribal territory of Gofa was situated approximately half the way “from Badditu to Dime” (see Fig. 1). For further discussion in this paper we treat this area as type locality of *L. gramineus*: Ethiopia, Gamu Gofa, 6°29'N, 36°59'E.

With the lectotype series, BMNH 1947.2.10.19–20 (2 specimens) and MSNG 28564A–B (2 specimens), at least the following specimens housed in museum collections should be considered as topotypic: BMNH 1975.1618–9 (2 specimens), 9 km N of Chencha, 27.6.1975, leg. Yalden and Largen; ZNHM H.558/1–2 (2 specimens), Chencha, 1971, leg. Rupp; ZNHM H.678/1–3 (3 specimens), Chencha, 1972, leg. Rupp; ZNHM H.634/1–2 (2 specimens), 1 km SW of Chencha, 9.9.1972, leg. Clarke; BMNH 1975.1633–4 (2 specimens), 4 km N of Dorse, 28.4.1972, leg. Yalden and Largen; BMNH 1969.193, Bonche Valley, 1969, leg. Sandhurst Ethiopia Expedition.

Since *L. gramineus* has never been reported from the Badditu territory, the other vernacular name of this species (found, for instance, in Largen and Spawls 2010; Channing and Rödel 2019) appears to be more appropriate: ‘Ethiopian Burrowing Treefrog’. The attribute ‘burrowing’ in the name reflects the established opinion that these frogs are fossorial (see Largen 1977; Schiøtz 1999; Largen 2001; Largen and Spawls 2010; Channing et al. 2012; Reyes-Velasco et al. 2018; Channing and Rödel 2019).

The confidence that our study sites do not lie in the distribution area of *L. gramineus* sensu stricto and available material allow a description of a new species.

#### Leptopelis diffidens sp. nov.

Taxon classificationAnimaliaAnuraArthroleptidae

D93D70FE-8092-5003-AC72-5A86F7058F7B

http://zoobank.org/E059D4EF-FFD4-40ED-B24F-A12AB7A998EF

Figures 2 , 3 , 4

##### Type locality.

Between rivers Welmel and Yadot, Harenna Forest, Bale Zone, Oromia Region, Ethiopia (6°35'N, 39°45'E).

##### Material.

***Holotype***: Ethiopia • ♀; Segoba glade, Harenna Forest, Bale Zone, Oromia Region; 6°35'10.5"N, 39°44'30.7"E, 1770 m a.s.l.; 1 June 2019; A. Tiutenko leg.; “Found in a tussock near calling male on a flooded forest glade during rainy season”; ZSM 81/2019. ***Paratypes***: Ethiopia • 1♂; Woraba glade, Harenna Forest, Bale Zone, Oromia Region; 6°35'39.9"N, 39°45'15.2"E, 1800 m a.s.l.; 5 June 2019, A. Tiutenko leg.; “Found in wet grass near slow flowing temporary stream in rainy season”; GenBank: MN909553; ZSM 83/2019 • 1 juvenile; Woraba glade, Harenna Forest, Bale Zone, Oromia Region; 6°35'39.3"N, 39°45'14.5"E, 1800 m a.s.l.; 5 June 2019; A. Tiutenko leg.; “Found in marshy grass about 10 m from a slowly flowing stream on a flooded glade”; ZSM 82/2019 • 1♀; Katcha, Bale Mts; 06°42'N, 39°44'E. 2400 m a.s.l.; 5 August 1986; Harenna Forest Expedition, M. J. Largen leg.; “Small stream near border between grassy clearing and dense Schefflera/Hagenia forest. Males calling from grass bordering stream”; LIV 1986.212.198 • 24 juveniles; Swamp near Shawe R., Bale Mts; 06°40'N, 39°44'E. 1980 m a.s.l.; 7 August 1986; Harenna Forest Expedition, M. J. Largen leg.; “Crawling up tall grasses surrounding swamp with open water and tall bushes, in Aningeria forest. Just metamorphosed juveniles, green and brown phases, latter with 3 longitudinal dark stripes, median confluent with interorbital bar”; LIV 1986.212.199–222.

##### Additional material.

Ethiopia • 1 larva; Woraba glade, Harenna Forest, Bale Zone, Oromia Region; 6°35'39.0"N, 39°45'14.6"E, 1800 m a.s.l.; 5 June 2019; A. Tiutenko leg.; “temporary puddle”; ZSM 172/2019.

##### Diagnosis.

Medium-sized (SVL of males ca. 24–29 mm, females ca. 35–40 mm) ground-dwelling and burrowing frog with robust body, relatively wide and short head, and short limbs. Terminal phalanges of toes and fingers expanded to small, but distinct discs. Only base phalanges of toes II–IV with broad web; on toe V the web extends along phalanges 1 and 2. The rest of toe phalanges with feeble fringe. Fingers are free of web or fringe. Light-brown or light olive-green from above; either no dorsal pattern, or three indistinct broad longitudinal bands (one vertebral and two dorsolateral) present that are slightly darker than the ground colour. Males with pectoral glands. Advertisement call: quiet, high-pitched singleton ‘quack’, repeated at intervals of ca. 20 seconds.

##### Description of holotype.

ZSM 81/2019, adult female. SVL 35.9 mm. The head slightly wider than its length (HW/HL 0.77), ca. 1/3 of the SVL (HL/SVL 0.32). Body oval. Eyes rather large (ED/HL 0.39, ED/SL 1.15, UEW/HW 0.20), positioned laterally (IOD 4.2 mm) and directed slightly forwards. Pupil vertical. Snout strongly curved downwards, especially in front of the nares. Canthus rostralis rounded. Naris approximately at half the distance between eye and snout tip. Tympanum visible, very close to posterior border of the orbit (ETD ca. 1.0 mm), comparatively small: TD 1.4 mm, TD/ED 0.31. Hind limbs rather short TL+THL/SVL 0.76. Tibio-tarsal articulation reaches to the anterior of the tympanum. Outer metatarsal tubercle absent. Inner metatarsal tubercle very large (IMT/TSL 0.24), compressed, ca. half as broad as long (IMTW/IMT 0.57). Terminal phalanges of toes and fingers with small, but rather conspicuous discs, e.g., Fin2W/Fin2DW 0.35, Fin4W/Fin2DW 0.67, Toe4W/Toe4DW 0.68. Fingers free of webbing. Toe I free of web. On toe II the web does not reach beyond phalanx 1. On toes III and IV it reaches to ca. 1/2 of the phalanx 2, and on toe V it even extends up to the joint of the phalanges 2 and 3. The web continues as a feeble fringe along the rest of the phalanges on all toes except toe I that is free of it. Dorsal skin finely granular, almost smooth, with scattered singleton tubercles. Feeble vomerine teeth form two small groups.

***Colouration in life*** pale green-brown or grey-green from above. Feeble one vertebral and two dorsolateral bands, a little darker than the ground colour, bordered with small irregular tubercles. The dorsal colour transits to light green and blue at thighs and shoulders. Venter off white, feebly mottled with grey. Gular area without pattern. Black band along canthus rostralis on each side of the head extending over the nostril to the eye. Behind the eye it continues over the tympanum and above the shoulder to approximately the middle of the flank. This band is not outlined. Iris dark bronze.

***Colouration in preservative***: Dorsum grey. Venter pale, grey mottled. Dorsolateral pattern as in life.

##### Variation of paratypes.

The paratype series comprises 27 specimens of various ages and sexes.

**Figure 2.**
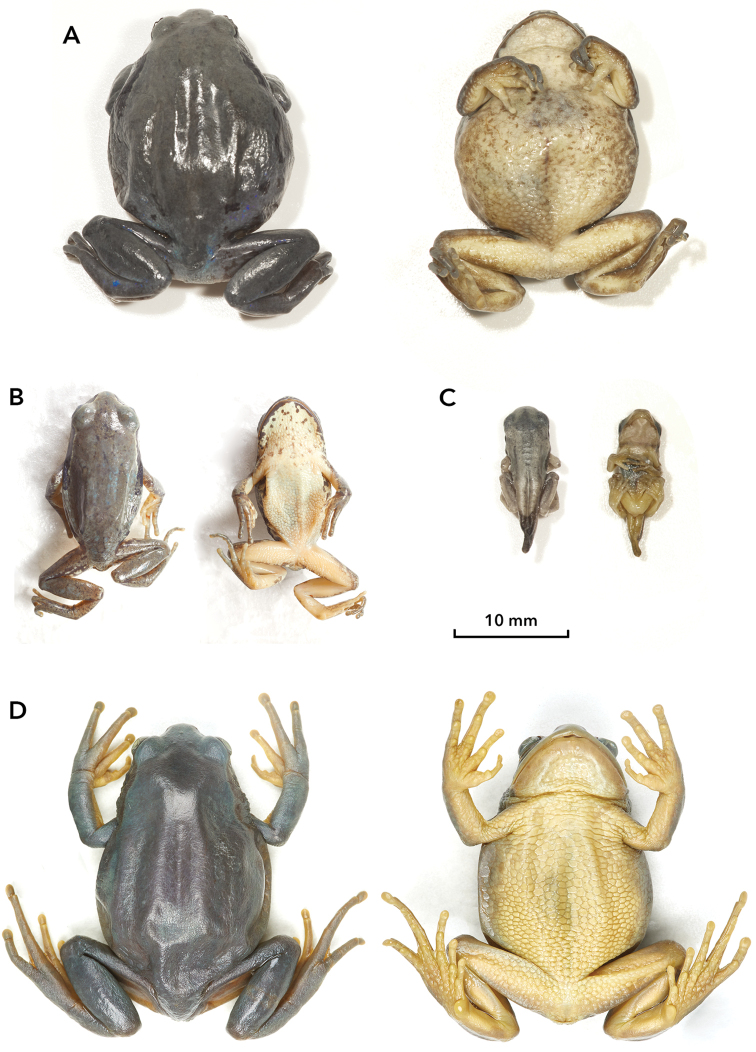
Types of *Leptopelis diffidens* sp. nov. (in dorsal and ventral view) **A** holotype, female, ZSM 81/2019 **B** paratype 1, male, ZSM 83/2019 **C** paratype 2, metamorph, ZSM 82/2019 **D** paratype 3, female, LIV 1986.212.198.

***Paratype 1*** (Fig. 2B): ZSM 83/2019, adult male. It has a much slenderer body than the holotype, whose body is almost round, and is much smaller than the female: SVL 21.8 mm versus 35.9 mm. The head is similarly large (HL/SVL 0.34), but even wider than in the female: HL/HW 0.88. Tympanum is of similar size: TD/ED 0.30. Pectoral glands visible. Digital discs are slightly larger: Fin2W/Fin2DW 0.51, Fin4W/Fin4TW 0.72, Toe4W/Toe4DW 0.77. Skin on all parts of the body and head smooth. Colouration in life: Dorsal colouration is very similar to that of the holotype: Pale olive-green from above, with three indistinct longitudinal bands. Ventral colouration is different and pale, without pattern. Throat with some scattered dark spots. Sides of thighs with dark grey blotches and bands. Similar pattern also between flanks and venter; this pattern is absent in the holotype. Similar to holotype, a dark band extends from snout tip, over the eye and above tympanum; however, it does not continue at both sides of the body beyond the shoulder. Unlike in holotype, there are no dark blotches on flanks. Colouration in preservative like in life but dorsal colour is grey.

***Paratype 2*** (Fig. 2C): ZSM 82/2019, metamorph at Gosner stage 45. Very small individual, SVL 12.9 mm, with tail stub – 5.9 mm. Head is similarly large in comparison to body as in adult individuals: HL/SVL 0.34. Other proportions are similar: HL/HW 0.89, ED/HL 0.46, ED/SL 0.95, TD/ED 0.30, TL+THL/SVL 0.63, IMT/TSL 0.29. The inner metatarsal tubercle is flatter than in adults: IMTW/IMT 0.25. Digital discs are similarly conspicuous in this stage of the life cycle: Fin2W/Fin2DW 0.83, Toe4W/Toe4DW 0.75. In life, the colouration was very similar to that of the male Paratype 1.

***Paratype 3*** (Fig. 2D): LIV 1986.212.198, adult female with even larger SVL than in the holotype – 39.6 mm, but with almost identical morphology and very similar appearance: HL/SVL 0.32, HL/HW 0.79, ED/HL 0.36, ED/SL 0.91, TD/ED 0.39, TL+THL/SVL 0.69, IMT/TSL 0.27, IMTW/IMT 0.99, Fin2W/Fin2DW 0.51, Fin4W/Fin4TW 0.72, Toe4W/Toe4DW 0.72. It is an old specimen that had been probably fixated or even kept for some time in formalin prior to current storage in alcohol; therefore it is hardly possible to compare its colouration with more recently collected and immediately ethanol preserved specimens. In its current state the dorsum of this specimen appears bluish-grey; the venter is yellowish. No pattern is recognisable, except dark lateral band which is similar to that in the holotype. Probably the live colour of dorsum of the Paratype 3 has been more uniformly green.

**Figure 3.**
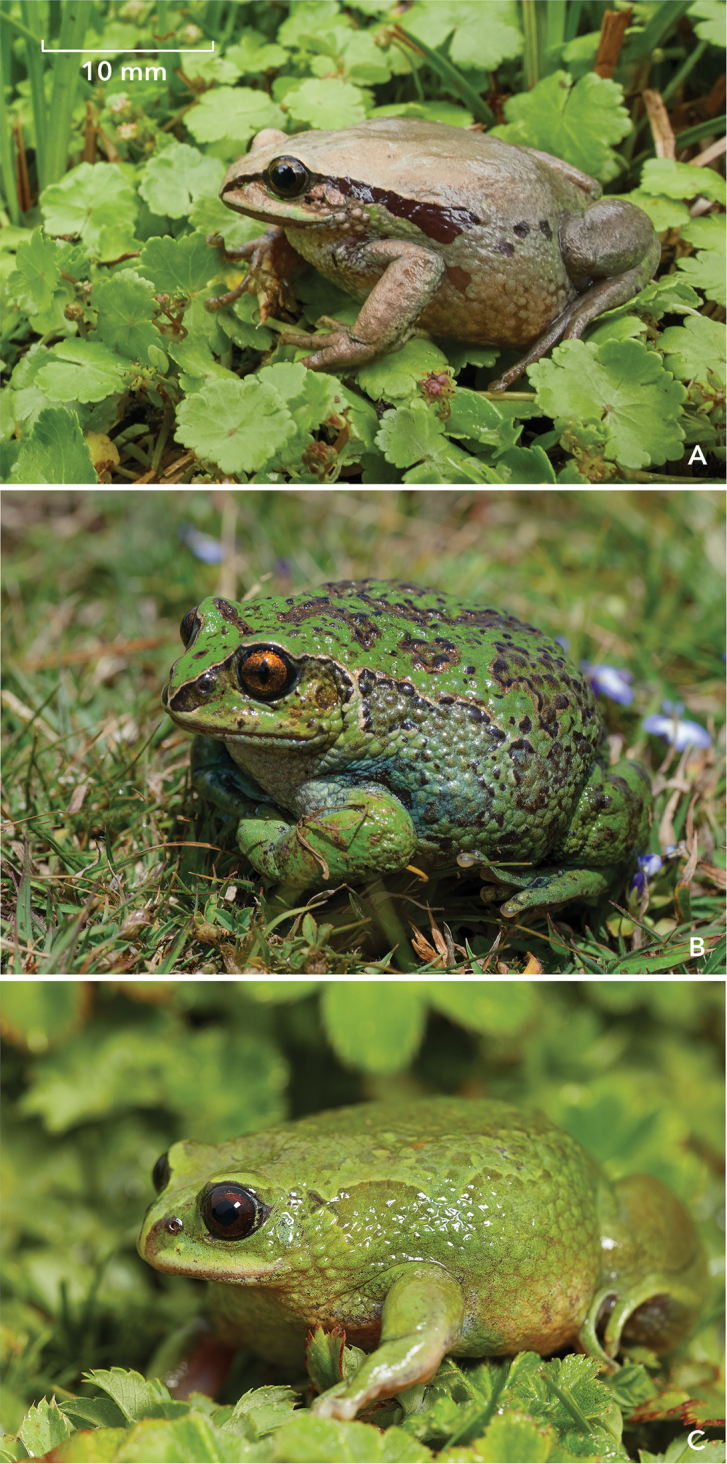
Adult females of three species of the *Leptopelis gramineus* complex in life, shown to the same scale **A***L. diffidens* sp. nov., Harenna Forest (holotype, ZSM 81/2019) **B***Leptopelis montanus* nom. nov., Gaysay Grasslands, Bale Mountains (not collected) **C***L.* sp. ‘Shewa’, Menz-Guassa (not collected).

**Figure 4.**
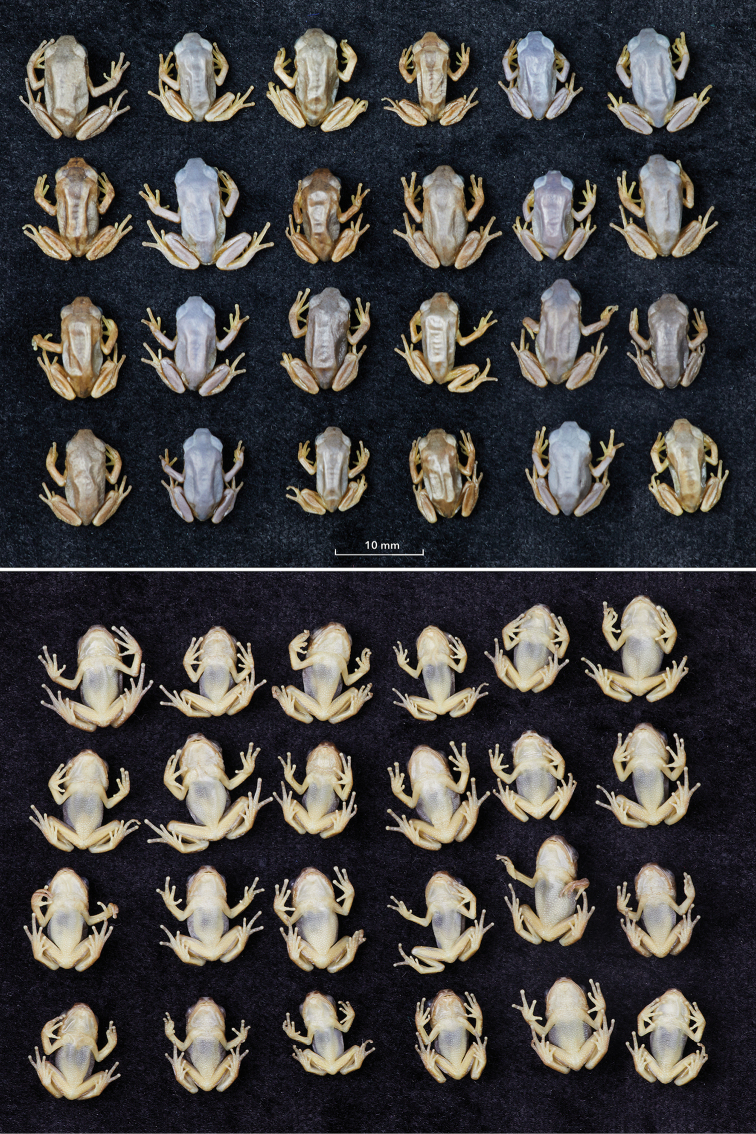
Paratypes 4–28, immature individuals, LIV 1986.212.199–222 (in dorsal and ventral views).

***Paratypes 4–27*** (Fig. 4): LIV 1986.212.199–222, recently metamorphosed individuals with SVL 11.1–14.5 mm (see Suppl. material 5: Table S1 for morphometrics of each specimen). They closely resemble the male Paratype 1 in body shape but are smaller and lack pectoral glands. In comparison with Paratype 2, they lack tails, i.e., have been at a more advanced development stage (Gosner stage 46) at time of collecting, though they are of similar size or just slightly larger. Like all other specimens of the type series, they have more or less continuous dark lateral bands extending from the snout tip to the middle of the flanks or further. Eight specimens appear rather bluish, compared to the rest of the series whose dorsal colour is brown. This may indicate that they were green in life. Among brown specimens some have also bluish areas which may have been green in life as well. The ventral side is uniformly pale, without any spots or pattern.

##### Differential diagnosis.

*Leptopelis diffidens* sp. nov. shares the following traits with the rest of the members of the genus *Leptopelis*: The terminal phalanx of finger kinked (out of alignment with the rest of phalanges); no fingers opposing each other; digital discs on fingers present; outer metatarsal tubercle absent; singleton subarticular tubercles; tympanum visible; pupil vertically elliptic; vomerine teeth arranged in two groups between choanae.

**Figure 5.**
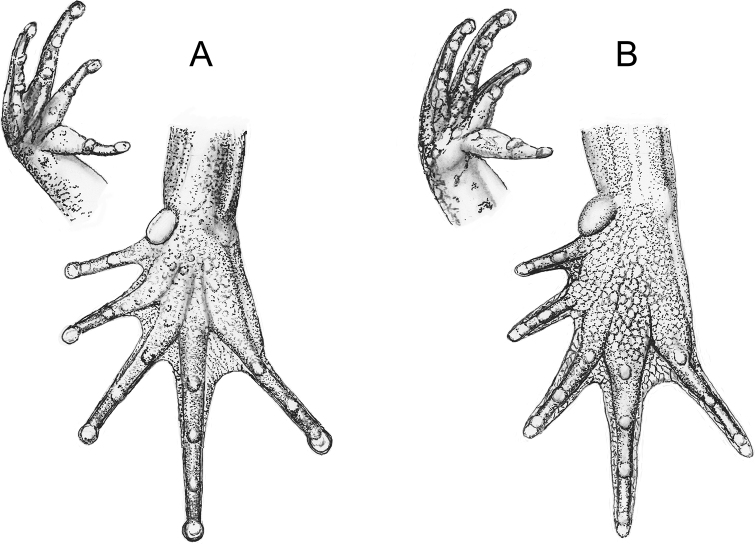
Comparison of digital discs (on right hand and foot) and of pedal webbing in **A***Leptopelis diffidens* sp. nov., and **B***L. montanus* nom. nov.

**Figure 6.**
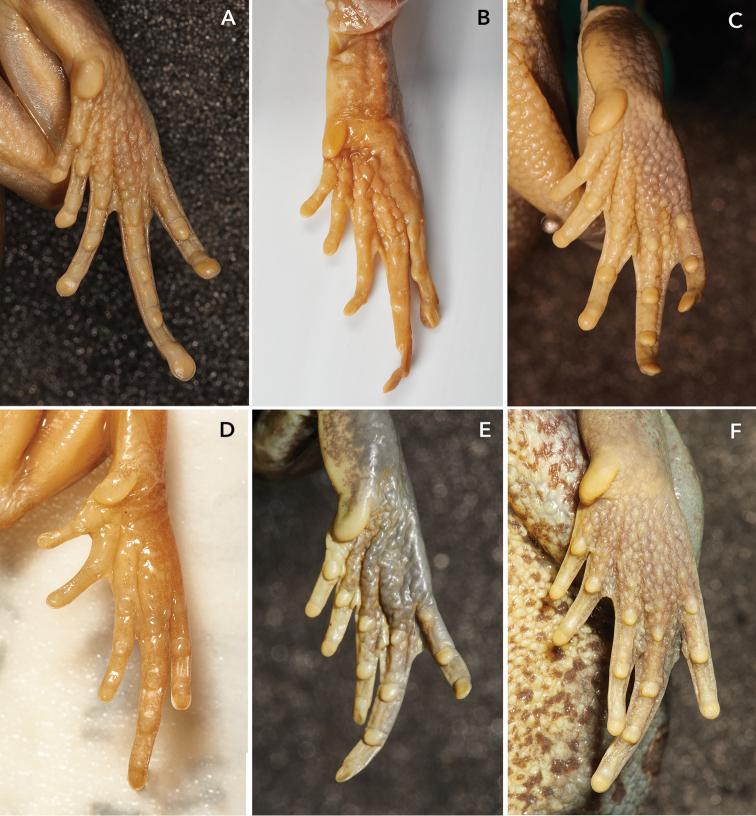
Pedal webbing of five members of the *Leptopelis gramineus* complex **A***L. diffidens* sp. nov., LIV 1986.212.198 **B***L. gramineus*, BMNH 1947.2.10.19 (paralectotype) **C***L. gramineus*, BMNH 1975.1619 **D***L.* sp. ‘Borana/Sidamo’, PEM 3821 **E***L. montanus* nom. nov., BMNH 1975.1621 **F***L.* sp. ‘Shewa’, BMNH 1969.969.

The digital discs both, on fingers and toes, are in the new species significantly more conspicuous than *L.* sp. ‘Shewa’ and in the parapartic high-altitude population in Bale and Arsi Mountains – *L. montanus* nom. nov. (Figs 5, 6). In the examined specimens of both high-altitude species the digital discs were virtually absent: On their fingers there are just feeble pads (Fin2W/Fin2DW 0.92, Fin4W/Fin4DW 0.86), and on toes the tips are even narrower than the phalanges (Toe4W/Toe4DW 1.8). The digital discs in *L. diffidens* sp. nov. are not significantly larger than in *L. gramineus* sensu stricto (Fig. 6). In two paralectotypes and five topotypic specimens that we measured the average ratio ‘disc width to phalanx width’ was 0.85 on finger II (Toe2W/Toe2DW) and 0.70 on toe IV (Toe4W/Toe4DW).

The examined males of both, *L.* sp. ‘Shewa’ and *L. montanus* nom. nov., are much larger than in the new species: SVL 27.2–40.6 mm (mean 35.6 mm). Females in these two species are even larger, with SVL 47.5–56.6 mm (mean 51.5 mm). Generally, females of *L. diffidens* sp. nov. are sized like males of the montane species in this species complex (see comparison photographs in Suppl. material 12).

All examined males of *L. gramineus* sensu stricto (three specimens, including paralectotype BMNH 1947.2.10.20) were significantly larger than of *L. diffidens* sp. nov.: mean SVL 36.1 mm; the size difference between male and female in *L. gramineus* appears to be small. Two of the examined three females (including paralectotype BMNH 1947.2.10.19) had SVL 39.72 mm and 39.37 mm. The third female (BMNH 1975.1633) with SVL 24.39 mm may be immature.

A principal component analysis showed that the low-altitude geographic group from the Harenna Forest that we describe here as *L. diffidens* sp. nov. is separated from the rest of the groups along the first axis accumulating 44.99% of morphological variation. The other groups are partially separated along the second axis accumulating 10.85% of variation while the most divergent groups are *L. gramineus* sensu stricto and the highland populations from the east of the GRV (*L. montanus* nom. nov.). The specimens from ‘Borana/Sidamo’ population are placed between them and partially overlap with both. See this plot in Suppl. material 10.

**Figure 7.**
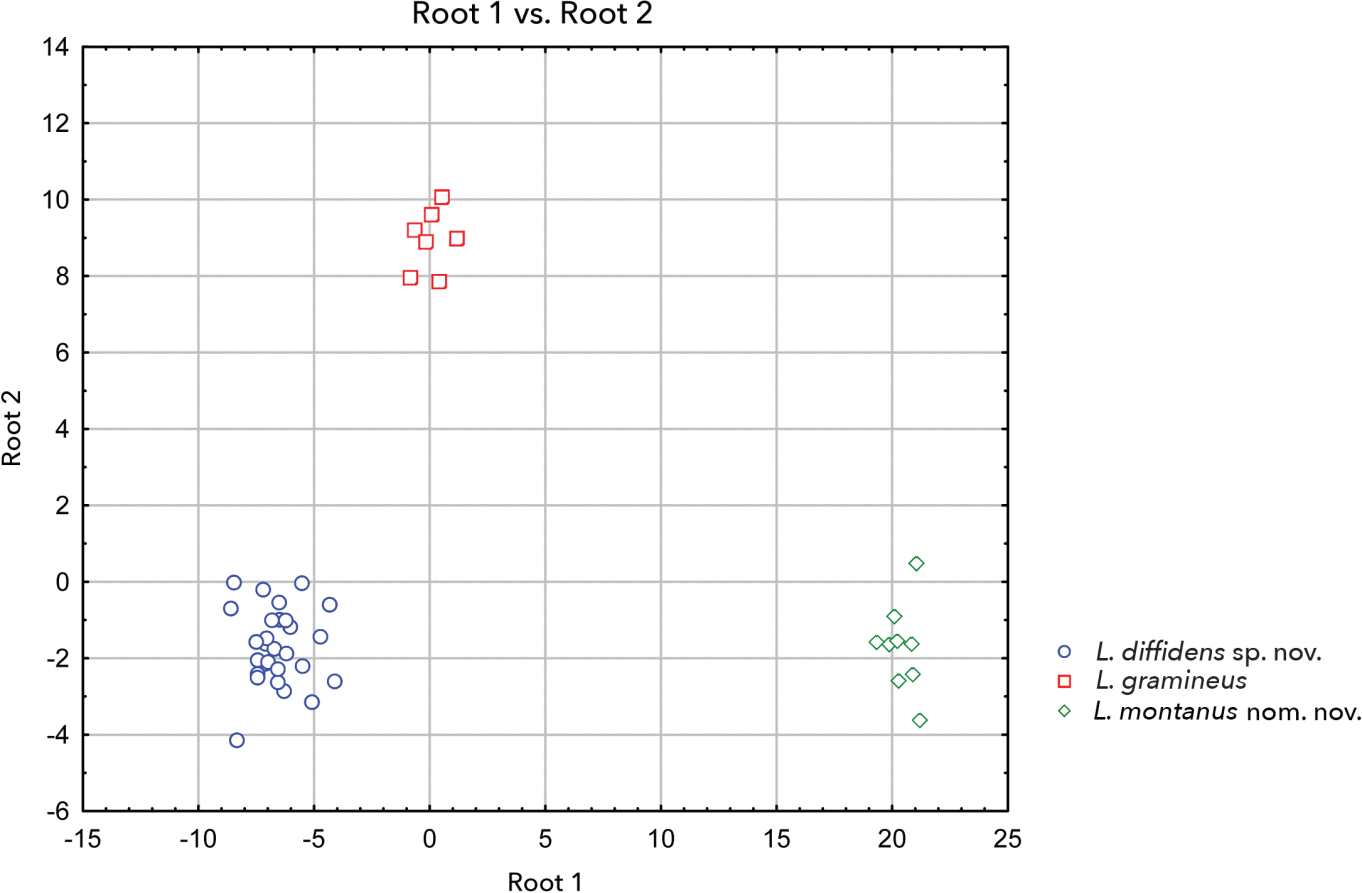
Grouping of three species of *Leptopelis gramineus* complex on a plane of two canonical roots. Discriminant analysis summary and standardised coefficients for canonical variables are provided in Suppl. material 1 and 3.

A canonical discriminant analysis separated the three groups *L. diffidens* sp. nov., *L. montanus* nom. nov., and *L. gramineus* sensu stricto with high confidence (see Fig. 7): Wilks’s Lambda approx. 0.00048, *F* (58, 26) = 20.111, *p* < .0000. Four measurements (HL, TL, Toe3L, Toe4L) had the largest contribution to the discriminant function. A discriminant analysis summary and standardised coefficients for canonical variables are provided in Suppl. material 1 and 3. In spite of moderate sample size and combining both sexes in a single multivariate analysis, we revealed strong differences in the body proportion between three lineages of the *L. gramineus* complex, manifested in head proportions, tibia, and longest toe lengths. As Lada et al. (2005) showed for European *Pelobates*, the fossorial way of life is a strong constraint for morphological variation and sexual dimorphism. Thus, the morphological divergence that we observed in the studied species of *L. gramineus* complex may be related to different extent of fossoriality.

Compared to all other frogs currently assigned to *L. gramineus*, the new species appears to have smoother skin on dorsum, with just some small singleton warts. *L. diffidens* sp. nov. differs from other members of the complex also in overall dorsal colouration: It is rather grey-green or pale beige while *L. gramineus* and other high-altitude species of this complex have more bright green or yellow-brown colouration. Furthermore, the new species has no such dorsal pattern of scattered blotches and ocelli as in the parapatric *L. montanus* nom. nov. The latter, in turn, usually lacks continuous black lateral bands that seems to be typical for *L. diffidens* sp. nov. (Fig. 3). Canthus rostralis in *L. diffidens* sp. nov. is not outlined light yellow or light green as in other members of this complex.

From non-sympatric, but also ground-dwelling, *L. bocagii* the new species differs by less robust body form, smaller head, rounded canthus rostralis (versus strongly angled in *L. bocagii*). The digital discs in *L. bocagii* are similarly expanded except on toes IV and V where they seem to be larger. The inner metatarsal tubercle is similarly large and compressed. Unlike in *L. bocagii* that has strong vomerine teeth, they are feeble in *L. diffidens* sp. nov. and additionally, the colouration of *L. bocagii* is very different, see Largen (1977) for a detailed description and illustration.

*Leptopelis diffidens* sp. nov. is generally ground-dwelling and does not occur on trees or scrubs, although males and juveniles may occasionally climb grass. From all scansorial members of the genus distributed at the Horn of Africa, including sympatric *L. ragazzii* (Boulenger, 1896), it differs by much more robust body (particularly in females), comparatively small head, shorter limbs, reduced digital discs. Moreover, *L. ragazzii* has a flatter head with less curved snout and comparatively larger eyes. This applies also to non-sympatric *L. vannutellii* (Boulenger, 1898), which does not differ morphologically from *L. ragazzii*. Both, *L. ragazzii* and *L. vannutellii*, have small inner metatarsal tubercle and well-developed webbing on feet, extending on toes III and IV well beyond the joint of the phalanges 1 and 2. Similarly to *L. ragazzii* that has two clearly different colour phases, bright green and brown, occurring together. *Leptopelis diffidens* sp. nov. exhibits such dichromatism as well, although not that distinct: unlike in *L. ragazzii*, the brown phase in the new species is not purely brown, but rather olive-green or grey-brown. No plain green phase was ever recorded, but grey-green or pale green individuals occur in populations of *L. vannutellii*. Overall, the colour of *L. vannutellii* is not like that of *L. diffidens* sp. nov.

The non-sympatric species *L. susanae* and *L. yaldeni* Largen, 1977 have much broader digital discs, small inner metatarsal tubercles, and more extensive webbing on toes III and IV than the new species. Both are scansorial forms with more slender bodies. *Leptopelis yaldeni* has two colour phases which are clearly distinct, green and brown, like in *L. ragazzii.* Similar to *L. diffidens* sp. nov., *L. susanae* does not show such clear dichromatism, but its general colouration clearly differs very much from the new species (compare photographs and description in Largen 1977).

Another species, *L. concolor* Ahl, 1929, recorded from the Juba river basin, south-east of the distribution area of *L. diffidens* sp. nov., is a scansorial form with a similar body size that occupies coastal savanna habitats. Similar to the new species, it has reduced pedal webbing but larger discs, particularly on toes III, IV, and V. Like other arboreal *Leptopelis*, it has a slender body and a large head. The colouration of *L. concolor* is rather constant: light brown from above, with dark interorbital bar and an indistinct reversed ‘Y’ on dorsum.

##### Advertisement call.

Males usually call from tussocks surrounded by water, but also from tall and dense grass at margins of pools and puddles, and occasionally from shrubs surrounding the glade. The animal sits near the roots of the grass, but not on grass and not in water. When approached, it stops calling and becomes silent for many minutes. The calls are rather weak and may be difficult to perceive in places with background chorus of other frogs, particularly of *Ptychadena*, or with loudly shouting birds. During a rainy season, in May–June, we did not notice any significant difference in the intensity of vocalisations between day and night. However, in the same season we observed more calling individuals and more frequent calls in the parapatric population of *L. montanus* nom. nov. in the Bale Mountains.

**Figure 8.**
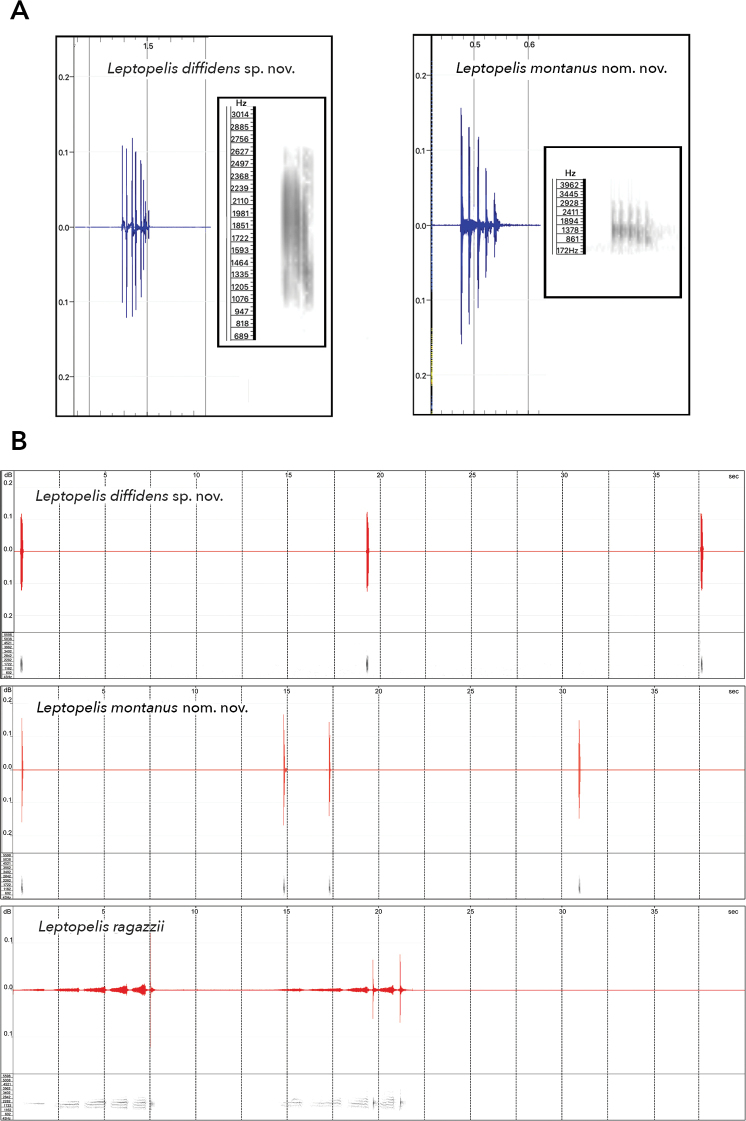
**A** comparison of singleton advertisement calls of *Leptopelis diffidens* sp. nov. and *L. montanus* nom. nov. **B** oscillograms (top) and spectrograms (bottom) of advertisement calls of *L. diffidens* sp. nov., *L. montanus* nom. nov.), and *L. ragazzii* within 40 seconds.

Like other Ethiopian members of this species complex (Largen 1977; Schiøtz 1999), the advertisement call of *L. diffidens* sp. nov. is a ‘quack’. It consists of a single note that contains eight pulses with interval of ca. 20 ms. The tone frequency is 990–2700 Hz (Fig. 8A). It is higher than in the call of the high-altitude member of the *L. gramineus* complex from the Bale Mountains that also consists of a single note, however, of five pulses and with a frequency of 344–3270 Hz (Fig. 8B). According to our observations, *L. diffidens* sp. nov. repeated their ‘quack’ with equal intervals of ca. 20 seconds. Another male may use the pause for his call. We did not observe two or more males calling synchronously. Largen (1977) mentioned a ‘scream’ that sometimes precedes the ‘quack’ in *L. gramineus* from high-altitude population (*L. montanus* nom. nov.). We did not observe such vocalisations in the new species at any localities we visited.

The vocalisation of *L. ragazzii* partially resembles that of *L. diffidens* sp. nov. in containing a ‘quack’ sound of similar pitch and frequency. However, it is normally preceded by a screech, lasting for ten or more seconds, that consists of 4 or 5 notes with increasing pitch and frequency (Fig. 8C). *L. diffidens* sp. nov. does not produce such sounds.

##### Genetics.

The 16S mitochondrial gene sequence (GenBank no. MN909554) of the specimen ZSM 83/2019 (Paratype 1) matches to 99.8% the corresponding sequences obtained by other workers from tissue samples of frogs that had been collected in the Harenna Forest at Katcha and in vicinity of Rira and identified as *L. gramineus*. Freilich et al. (2016) applied phylogenetic reconstructions based on the COI gene and found four highly divergent mitochondrial lineages in *L. gramineus* with average distance of 6.4–8.0% between them. They restricted three of these clades to the east of the GRV and called ‘Arsi’, ‘Kibre Mengist’ and ‘Kasha’. These correspond to what we call here *L. montanus* nom. nov., *L.* sp. ’Borana/Sidamo’, and *L. diffidens* sp. nov. (see Suppl. material 8: Table S4).

As Reyes-Velasco et al. (2018) showed with their genetic study using multiple RAD loci, the population from the Harenna Forest and the related population from Bore and Kibre Mengist area (*L.* sp. ‘Borana/Sidamo’) represent together a sister clade of *L. susanae*. This lineage diverged from the high-altitude species *L. montanus* nom. nov. around 4 my ago, during the Pliocene. The split between eastern and western lineages of the *L. gramineus* complex took place ca. 6 my ago. Therefore, the new species from the Harenna Forest is more distantly related to species from the west of the GRV, hence also to *L. gramineus* sensu stricto.

**Figure 9.**
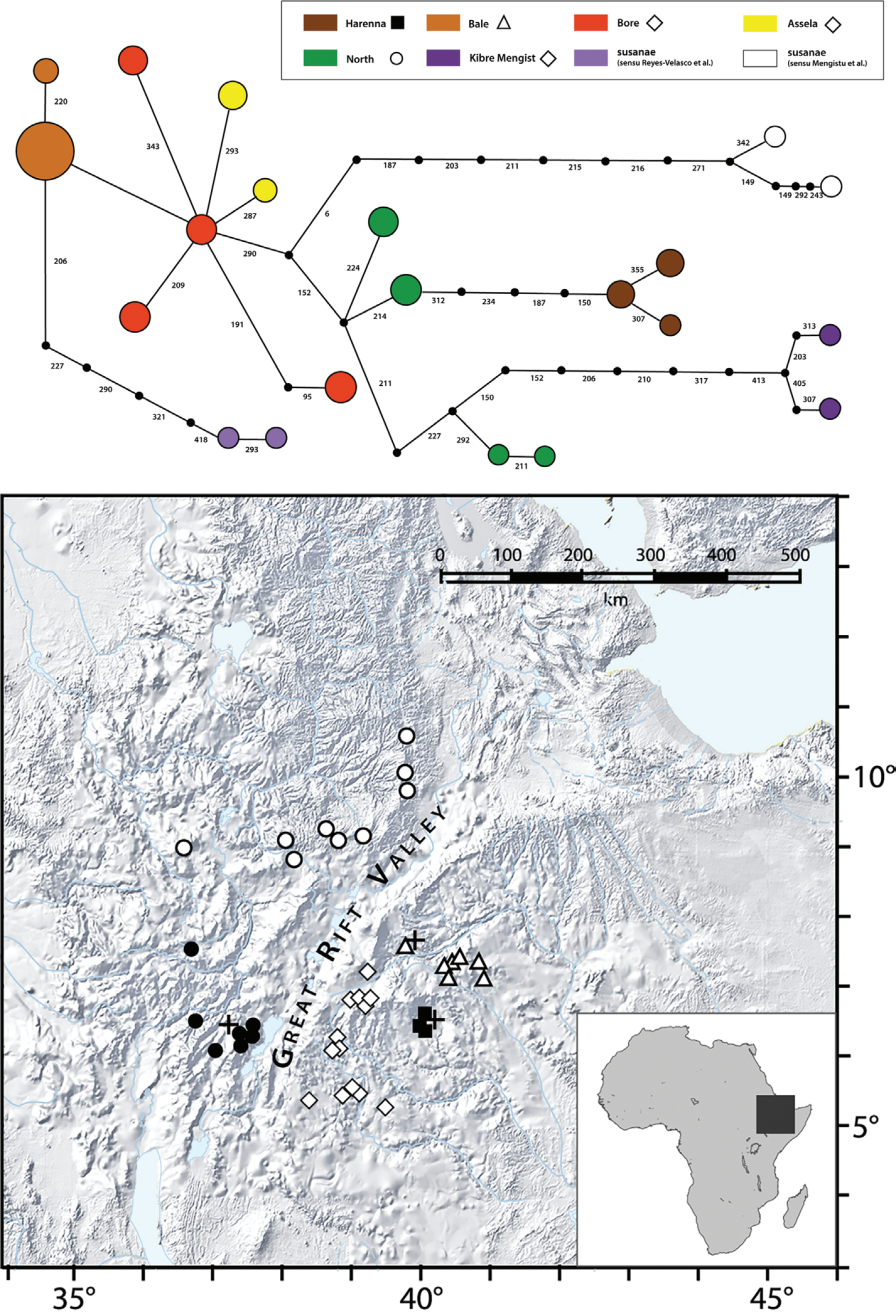
Network of haplotypes and a map showing the distribution of the *Leptopelis gramineus* species complex members: *L. gramineus* (filled circle), *L.* sp. ‘Shewa’ (empty circle), *L. montanus* nom. nov. (empty triangle), *L. diffidens* sp. nov. (filled square), *L.* sp. ‘Borana/Sidamo’ (empty diamond). Type localities of the described species are marked with a cross. The colours on the haplotypes chart correspond to various groups recognised by Mengistu et al. (2012), Freilich et al. (2016) and Reyes-Velasco et al. (2018). The symbols in the legend correspond to populations shown on the map.

In order to align our material to the already defined groups we have constructed a statistical parsimony network of 16S haplotypes (showed in Fig. 9). Both, the paratype of *L. diffidens* sp. nov. (GenBank no. MN909554) and the sequenced specimen from the Gaysay Grasslands (GenBank no. MN909551) clustered respectively, with the representatives of the geographic groups of the low-altitude Harenna Forest (i.e., clades ‘Harenna’ sensu Reyes-Velasco et al. 2018 and ‘Kasha’ sensu Freilich et al. 2016) and of the high-altitude population that Freilich et al. (2016) called ‘Arsi’ and Reyes-Velasco et al. (2018) called ‘Bale Mountains’.

##### Etymology.

The Latin adjective *diffidens* (used here in nominative singular) means diffident, anxious, shy, mistrustful. This name refers to the behaviour of this frog that appears even more cryptic and defensive than *L. gramineus* from other populations. The vocalisation of a male is so quiet that the animal is very difficult to locate and to spot. When approached, at a distance of only 5 m, it stops calling and remains silent until the disturber has gone away or has not moved for at least 10 minutes. As a vernacular name for this species, we propose ‘Harenna Burrowing Treefrog’.

##### Distribution and habitat.

According to our current knowledge, the new species is endemic to the Harenna Forest where it occurs at elevations of 1400–2300 m a.s.l. We observed these frogs and recorded vocalisations at the following localities, which are glades in a tall forest: Gola, Hacho, Harawa, Haro Alati, Hordoba, Kaffa Guasaa, Manyate, Megano, Segoba, Sire, Woraba, and Yagana (Fig. 9). This species was also collected many times from Katcha and nearby localities.

*Leptopelis diffidens* sp. nov. seems to be confined to glades in the forest that are temporarily flooded with rain water during rainy season. These frogs do not live in areas of dense high trees, where they are replaced by *L. ragazzii*, typically found along streams. These two species never occur syntopically. We also did not encounter *L. diffidens* sp. nov. in man-made open areas in the forest, such as clearings, plantations, or settlements. However, we found two males of *L. ragazzii* (brown and green phase) on Aboye, a large clearing regularly used for barley plantation. Moreover, unlike *L. ragazzii* and the highland members of the *L. gramineus* complex whose larvae live in fast-flowing water, the new species uses lentic or slow-flowing waterbodies for breeding and does not occur at fast flowing streams (Fig. 9). We assume therefore that this frog requires a certain flora composition and environmental, particularly hydrological, conditions that are available only in natural glades.

*Leptopelis diffidens* sp. nov. shares breeding sites with *Phrynobatrachus inexpectatus*, *Ptychadena* sp., and *Xenopus clivii*. We observed this even in the middle of Manyate, a large village in the south of the Harenna Forest, because it has been built around a natural glade that retained its flora and hydrological characteristics.

An altitudinal and ecological barrier exists between *L. diffidens* sp. nov. and the not-yet-named montane grassland-dwelling *L. montanus* nom. nov.: *L. diffidens* sp. nov. does not ascend the Harenna Escarpment and is absent already in the sub-alpine heathlands, i.e., above 2500 m a.s.l. On the other hand, *L. montanus* nom. nov. that is distributed in the high-altitude areas, including the Sanetti Plateau, was not recorded in the areas directly adjacent to the Harenna Escarpment that have elevations around 4000 m a.s.l.; hence, no contact zone seems to exist here (Fig. 9). *Leptopelis diffidens* sp. nov. may not range west beyond the Ladamo mountain chain because suitable glades are absent there. This ridge also seems to isolate *L. diffidens* sp. nov. from populations of *L.* sp. ‘Borana/Sidamo’, a not-yet-named sister clade which was reported from localities further west, in Borana and in northern Sidamo (Reyes-Velasco et al. 2018). In the east, the range of *L. diffidens* sp. nov. reaches into Berbere area of the Harenna Forest where the forest gradually transits to bushland and savanna. The southern range boundary seems to be at Manyate and Haro Alati glade. South of this line glades disappear and the forest merges with Acacia bushland.

##### Sexual characters.

Males can be distinguished from females by a much smaller body size, less robust body, larger head, and the presence of pectoral glands.

##### Natural history.

*Leptopelis diffidens* sp. nov. is a ground-dwelling and semi-fossorial frog that spends most of its life hidden. In the dry season it is nocturnal, but in the rainy season we did not notice a significant difference between diurnal and nocturnal activity patterns at least in males.

Since we found a gravid female of *L. diffidens* sp. nov. and individuals at the last stages of metamorphosis on the same day and at the same locality, we assume that this species breeds more than once a year. Its breeding seasons appear to be out of sync with that of the parapatric high-altitude population of *L. montanus* nom. nov. in the Bale Mountains. We found metamorphs of *L. diffidens* sp. nov. (ZSM 82/2019 and ZSM 172/2019) in June, at the end of the short rainy season. A few days earlier we had visited a population of *L. montanus* nom. nov. in the Gaysay Grasslands and found neither metamorphs nor larvae. The rainy season there had just begun. In the Web Valley, Bale Mountains, we observed large tadpoles of *L. montanus* nom. nov. in the middle of February, at the end of a dry season. However, just a week later at Katcha in the Harenna Forest we found all puddles dried, and no frogs were around. During another trip, we found tadpoles in advanced development stages and metamorphs of *L. montanus* nom. nov. in vicinity of Dinsho village, Bale Mountains, in early November but saw neither adults nor larvae of *L. diffidens* sp. nov. after that in the Harenna Forest where the rainy season was approaching its end.

##### Larva.

The tadpole of *L. gramineus* has been described by Largen (1977) based on specimens from high-altitude populations and compared with the tadpole of sympatric *L. ragazzii* by Tiutenko and Zinenko (2019). Considering the morphological similarity and close phylogenetic relationship of *L. diffidens* sp. nov. we expect the tadpole of the new species to be similar to the tadpoles in the rest of this species complex. Except for one specimen ZSM 172/2019 at Gosner stage 43, no larvae of *L. diffidens* sp. nov. have been collected so far. This specimen was at time of collection shortly before leaving water and resembles an adult *L. diffidens* sp. nov. closely enough to permit positive identification. It has completely developed forelimbs. Instead of larval mouthparts it has an almost completely developed oral cavity and even a tongue. However, the development of the mouth opening is incomplete: it is only 2.4 mm wide, oval, and directed forward. Additionally, the tympanum is not yet present. All measurements are a little larger than those of another specimen (ZSM 82/2019) that we found at the same locality in a more advanced metamorphotic stage (see Suppl. material 5: Table S1).

##### Status of other cryptic species in *Leptopelis gramineus* complex.

Largen (1977) pointed out in his review of the Ethiopian *Leptopelis* that there is a “*great variation of size which has been demonstrated between, but not within, populations*” of *L. gramineus*. Since his so-called ‘populations’ are now treated as cryptic species, the pattern of these size differences has become clearer. The high-altitude species are generally considerably larger than the low-altitude ones. The 12 small male specimens from Wando (BMNH 1973.2164-2176) that Largen mentions are probably of the species that we call here *L.* sp. ‘Borana/Sidamo’ which is closely related to *L. diffidens* sp. nov. that is characterised by the same ‘dwarfism’. The largest (BMNH 1975.1638) of the 27 males examined of this species measured 29.6 mm. The largest of four females examined measured 40.0 mm. The mean SVL values are 25.4 (males) and 38.6 mm (females).

The population at the high-altitude lake Wonchi that Largen also mentions as an example of small-sized *L. gramineus* requires an additional study. Unfortunately, we did not find the specimens that Largen refers to (AAU H.548/1–2 and AAU H.718/1–4) during our visit in the ZNHM. Geographically this population should be within the range of *L.* sp. ‘Shewa’, and the locality is situated at high elevation of almost 3500 m a.s.l. This was also confirmed by DNA barcoding performed by Mengistu et al. (2012). Whether small body size is typical for this population, is yet to be determined.

For resolving the taxonomic status of the western populations of *L. gramineus*, new material and DNA samples from the type locality or from nearby highlands in Ethiopian administrative zones Gamo Gofa, Keffa, Wolayita, Kenta, and Dawuro are required together with further studies of morphological differences, bioacoustics, ecology, and contact zones. Mengistu et al. (2012) mentioned tissue samples of specimens from Gamo Gofa, but did not report any further results of their analysis. Other workers (Freilich et al. 2016; Reyes-Velasco et al. 2018) did not include topotypic material in their published results. Reyes-Velasco et al. (2018) had obtained nuclear DNA sequences of vouchers from Dorse and Chencha (Gamo Gofa), and from Bonga (Keffa), places at and close to the type locality, but apparently did not use them in their analysis for some reason. Jimma is the geographically closest locality from which DNA sequences were analysed so far. Reyes-Velasco et al. (2018) found that this population is rather different from *L. gramineus* further north, around Addis Ababa, Wonchi, and at other localities in Shewa and Gojam (*L.* sp. ‘Shewa’). We compared specimens of *L. gramineus* sensu stricto (paralectotypes and topotypic specimens housed in BMNH) with specimens from the north and found considerable difference between these populations in digital discs size, skin surface structure, body size and colouration. Therefore, it appears likely that the ‘northern’ population (*L.* sp. ‘Shewa’) represents a different species. Ahl (1924: 9) described a new species *Pseudocassina rugosa*, with type locality in Meta. In his later work he assigned it to the genus *Leptopelis* (Ahl 1931: 222) without any explanation. Largen synonymised *L. rugosus* with *L. gramineus* in the course of a revision of Ethiopian *Leptopelis* (Largen 1977). If comparison of genetic, acoustic, and morphological data with topotypic material would confirm a sufficient level of divergence, reinstatement of *Leptopelis rugosus* (Ahl, 1924) may be considered. Also, it is not clear how far this putative species ranges southwards, so there is no confidence that it does reach the type territory of *L. gramineus*.

**Figure 10.**
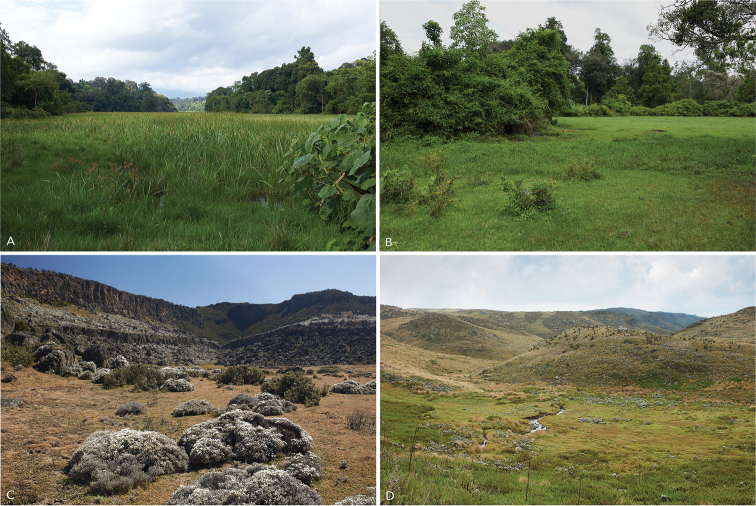
**A, B** Habitats of *Leptopelis diffidens* sp. nov. in the Harenna Forest – Segoba glade and Manyate village **C** habitat of *L. montanus* nom. nov. in the Web Valley, Bale Mountains **D** habitat of *L.* sp. ‘Shewa’ in Menz-Guassa.

In the same paper Ahl (1924) described *Pseudocassina ocellata*, another species that Largen (1977) synonymised with *L. gramineus*. Its type locality is Dida Plateau (‘Hochebene Didda’) which he first erroneously placed in Somalia, as he did with some other specimens collected by Neumann and Erlanger (see discussion in Largen 1975). This plateau is in fact situated in the Arsi Mountains, and the *Leptopelis* population there is certainly conspecific with frogs from other localities in Arsi and Bale. According to the published genetic data, this clade is reproductively isolated from other lineages of *L. gramineus* complex to the west from the GRV and could have limited exchange of genetic material with Kofele lineages sensu Reyes-Velasco et al. (2018). Therefore, we resurrect this species from synonymy and raise *L. ocellatus* (Ahl, 1924) to the status of a valid species in genus *Leptopelis*. As the vernacular English name we propose ‘Ocellated Burrowing Treefrog’ because it describes the typical colouration of this animal and matches the original description by Ahl (1924). However, the Latin name *ocellatus* is not available in this genus because a valid species from West Africa was described earlier and now bears the same name: *L. ocellatus* (Mocquard, 1902). In accordance with Article 50 and Paragraph 3 of Article 60 of ICZN, we establish a substitute name *Leptopelis montanus* nom. nov. for *Leptopelis ocellatus* (Ahl, 1924) to resolve its homonymy with *Leptopelis ocellatus* (Mocquard, 1902).

##### Etymology.

The new specific name *montanus* (living in mountains, montane) refers to the fact that this species ranges into afromontane areas up to elevation of almost 4000 m a.s.l., thus being a *Leptopelis* with probably the highest altitudinal distribution. The name is an adjective in nominative singular.

### Key to the Ethiopian species of the genus *Leptopelis*

This key covers only species of the genus that are currently known from Ethiopia and were formally described. Species candidates are not included due to lack of knowledge about their morphological traits.

**Table:** 

1	Metatarsal tubercle small, length not exceeding 6% of SVL	**2**
–	Metatarsal tubercle large, length ca. 8% of SVL	**5**
2	Tibia long, ca. 50% of SVL or longer	**3**
–	Tibia moderate, shorter than 50% of SVL	**4**
3	Blue-green colouration at axilla and groin	*** vannutellii ***
–	No blue-green colouration at axilla and groin	*** ragazzii ***
4	Dorsal pattern (dark triangle) not confluent with interorbital bar	*** yaldeni ***
–	Dorsal pattern (mid-dorsal stripe) confluent with interorbital bar	*** susanae ***
5	Canthus rostralis strongly angled	*** bocagii ***
–	Canthus rostralis rounded	**6**
6	Digital discs distinct, at least 30% wider than terminal phalanx	**7**
–	Digital discs indistinct, not wider than terminal phalanx	***montanus* nom. nov.**
7	Dorsal skin smooth, with small singleton warts	***diffidens* sp. nov.**
–	Dorsal skin rough	*** gramineus ***

## Supplementary Material

XML Treatment for Leptopelis diffidens

## Supplementary materials

Supplementary material 1 Discriminant function analysis summary Data type statistics Explanation note Supplementary data of multivariate canonical discriminant analysis. https://binary.pensoft.net/file/519114This dataset is made available under the Open Database License (http://opendatacommons.org/licenses/odbl/1.0/). The Open Database License (ODbL) is a license agreement intended to allow users to freely share, modify, and use this Dataset while maintaining this same freedom for others, provided that the original source and author(s) are credited.Oleksandr Zinenko

Supplementary material 2 Factor Structure Matrix Data type statistics Explanation note Supplementary data of multivariate canonical discriminant analysis. https://binary.pensoft.net/file/519115This dataset is made available under the Open Database License (http://opendatacommons.org/licenses/odbl/1.0/). The Open Database License (ODbL) is a license agreement intended to allow users to freely share, modify, and use this Dataset while maintaining this same freedom for others, provided that the original source and author(s) are credited.Oleksandr Zinenko

Supplementary material 3 Standardized Coefficients Data type statistics Explanation note Supplimentary data of multivariate canonical discriminant analysis. https://binary.pensoft.net/file/519116This dataset is made available under the Open Database License (http://opendatacommons.org/licenses/odbl/1.0/). The Open Database License (ODbL) is a license agreement intended to allow users to freely share, modify, and use this Dataset while maintaining this same freedom for others, provided that the original source and author(s) are credited.Oleksandr Zinenko

Supplementary material 4 Mean plots of morphometric characters grouped by species Data type statistics Explanation note Supplimentary data of multivariate canonical discriminant analysis. https://binary.pensoft.net/file/519117This dataset is made available under the Open Database License (http://opendatacommons.org/licenses/odbl/1.0/). The Open Database License (ODbL) is a license agreement intended to allow users to freely share, modify, and use this Dataset while maintaining this same freedom for others, provided that the original source and author(s) are credited.Oleksandr Zinenko

Supplementary material 5 Table S1. Morphometric measurements of specimens from three populations of *Leptopelis gramineus* complex Data type morphological Explanation note The table contains morphometric data from 45 specimens found in collections., including the holotype and the paratypes of *L. diffidens* sp. nov. For meaning of abbreviations see Material and methods in the main article text. https://binary.pensoft.net/file/519118This dataset is made available under the Open Database License (http://opendatacommons.org/licenses/odbl/1.0/). The Open Database License (ODbL) is a license agreement intended to allow users to freely share, modify, and use this Dataset while maintaining this same freedom for others, provided that the original source and author(s) are credited.Arthur Tiutenko

Supplementary material 6 Table S2. Examined material Data type specimen list Explanation note List of examined 105 specimens of Leptopelis gramineus complex and 86 specimens of six other Leptopelis species occuring sympatrically or in nearby geographic areas. https://binary.pensoft.net/file/519119This dataset is made available under the Open Database License (http://opendatacommons.org/licenses/odbl/1.0/). The Open Database License (ODbL) is a license agreement intended to allow users to freely share, modify, and use this Dataset while maintaining this same freedom for others, provided that the original source and author(s) are credited.Arthur Tiutenko

Supplementary material 7 Table S3. Gazetteer Data type geographic Explanation note List of geographic locations mentioned in the text of the article with their geographic coordinates and elevation. https://binary.pensoft.net/file/519120This dataset is made available under the Open Database License (http://opendatacommons.org/licenses/odbl/1.0/). The Open Database License (ODbL) is a license agreement intended to allow users to freely share, modify, and use this Dataset while maintaining this same freedom for others, provided that the original source and author(s) are credited.Arthur Tiutenko

Supplementary material 8 Table S4. GenBank sequences Data type phylogenetic Explanation note This table contains accession numbers of all mtDNA (16S) sequences used for this study that are published in GenBank along with names of clades (given by the authors) that these sequences belong to. https://binary.pensoft.net/file/519121This dataset is made available under the Open Database License (http://opendatacommons.org/licenses/odbl/1.0/). The Open Database License (ODbL) is a license agreement intended to allow users to freely share, modify, and use this Dataset while maintaining this same freedom for others, provided that the original source and author(s) are credited.Arthur Tiutenko

Supplementary material 9 Data table of geographic locations Data type geographic Explanation note Gazetteer in a software readable format. https://binary.pensoft.net/file/519122This dataset is made available under the Open Database License (http://opendatacommons.org/licenses/odbl/1.0/). The Open Database License (ODbL) is a license agreement intended to allow users to freely share, modify, and use this Dataset while maintaining this same freedom for others, provided that the original source and author(s) are credited.Arthur Tiutenko

Supplementary material 10 Principal component analysis plot Data type statistics Explanation note Projection of the cases on the factor-plane (1 × 2). https://binary.pensoft.net/file/519123This dataset is made available under the Open Database License (http://opendatacommons.org/licenses/odbl/1.0/). The Open Database License (ODbL) is a license agreement intended to allow users to freely share, modify, and use this Dataset while maintaining this same freedom for others, provided that the original source and author(s) are credited.Oleksandr Zinenko

Supplementary material 11 Phylogenetic tree Data type phylogenetic Explanation note Phylogenetic tree built from 16S mtDNA sequences. https://binary.pensoft.net/file/519124This dataset is made available under the Open Database License (http://opendatacommons.org/licenses/odbl/1.0/). The Open Database License (ODbL) is a license agreement intended to allow users to freely share, modify, and use this Dataset while maintaining this same freedom for others, provided that the original source and author(s) are credited.Oleksandr Zinenko

Supplementary material 12 Comparison of mature female *Leptopelis diffidens* sp. nov. and mature male *L. montanus* nom. nov. Data type multimedia Explanation note Adult females of the new speices have a similar size as adult males of the parapatric species from Bale Mountains. This plate allows visual comparison of two specimens. https://binary.pensoft.net/file/519125This dataset is made available under the Open Database License (http://opendatacommons.org/licenses/odbl/1.0/). The Open Database License (ODbL) is a license agreement intended to allow users to freely share, modify, and use this Dataset while maintaining this same freedom for others, provided that the original source and author(s) are credited.Arthur Tiutenko
