# Supplementary material for: A new species of Leptopelis (Anura, Arthroleptidae) from the south-eastern slope of the Ethiopian Highlands, with notes on the Leptopelis gramineus species complex and the revalidation of a previously synonymised species
Source: Zookeys. 2021 Mar 11;1023:119–50. doi: 10.3897/zookeys.1023.53404 (PMC7973069; doi:10.3897/zookeys.1023.53404)
Supplement: Supplementary material 1 — Discriminant function analysis summary [file zookeys-1023-119-s001.pdf]

N=44	Discriminant Function Analysis Summary (Leptopelis_all.sta)<br>No. of vars in model: 29; Grouping: sp (3 grps)<br>Wilks' Lambda: .00048 approx. F (58,26)=20.111 p< .0000					
	Wilks' Lambda	Partial Lambda	F-remove (2,13)	p-level	Toler.	1-Toler. (R-Sqr.)
HW	0.0006	0.77	1.96	0.18	0.16	0.84
HL	*0.0008*	*0.58*	*4.73*	*0.03*	*0.09*	*0.91*
ED	0.0005	0.98	0.14	0.87	0.08	0.92
EN	0.0005	0.95	0.35	0.71	0.03	0.97
NS	0.0005	0.94	0.39	0.68	0.03	0.97
SL	0.0005	0.95	0.31	0.74	0.26	0.74
IOD	0.0005	0.97	0.21	0.81	0.11	0.89
UEW	0.0005	0.98	0.11	0.89	0.08	0.92
IND	0.0005	0.89	0.79	0.47	0.16	0.84
TD	0.0006	0.77	1.99	0.18	0.03	0.97
FLL	0.0005	0.93	0.47	0.63	0.05	0.95
Fin1L	0.0005	0.98	0.15	0.86	0.22	0.78
Fin2L	0.0005	0.90	0.73	0.50	0.14	0.86
Fin2W	0.0006	0.77	1.95	0.18	0.08	0.92
Fin2DW	0.0005	0.89	0.80	0.47	0.08	0.92
Fin3L	0.0007	0.69	2.87	0.09	0.11	0.89
Fin4L	0.0007	0.66	3.31	0.07	0.09	0.91
Fin4DW	0.0006	0.74	2.29	0.14	0.07	0.93
TL	*0.0008*	*0.59*	*4.49*	*0.03*	*0.03*	*0.97*
THL	0.0005	0.96	0.30	0.74	0.03	0.97
TSL	0.0007	0.68	3.06	0.08	0.04	0.96
Toe1L	0.0006	0.82	1.40	0.28	0.07	0.93
Toe2L	0.0005	0.91	0.65	0.54	0.08	0.92
Toe3L	*0.0008*	*0.62*	*3.93*	*0.05*	*0.06*	*0.94*
Toe4L	*0.0008*	*0.63*	*3.85*	*0.05*	*0.06*	*0.94*
Toe4W	0.0005	0.96	0.28	0.76	0.10	0.90
Toe4DW	0.0005	0.99	0.07	0.93	0.09	0.91
Toe5L	0.0005	0.92	0.53	0.60	0.04	0.96
IMT	0.0005	0.99	0.09	0.92	0.03	0.97
