# Supplementary material for: A new species of Leptopelis (Anura, Arthroleptidae) from the south-eastern slope of the Ethiopian Highlands, with notes on the Leptopelis gramineus species complex and the revalidation of a previously synonymised species
Source: Zookeys. 2021 Mar 11;1023:119–50. doi: 10.3897/zookeys.1023.53404 (PMC7973069; doi:10.3897/zookeys.1023.53404)
Supplement: Supplementary material 2 — Factor Structure Matrix [file zookeys-1023-119-s002.pdf]

Variable	Factor Structure Matrix (Leptopelis_all.sta) Correlations Variables - Canonical Roots (Pooled-within-groups correlations)	
	Root 1	Root 2
HW	-0.093173	0.023618
HL	-0.053072	-0.184055
ED	-0.071223	-0.192621
EN	0.151415	0.094993
NS	0.138229	0.116171
SL	0.023944	-0.013981
IOD	-0.102943	-0.044556
UEW	-0.022159	-0.106530
IND	-0.015963	-0.184318
TD	0.178100	0.218611
FLL	0.035292	-0.017641
Fin1L	0.112157	0.123302
Fin2L	0.005539	0.174156
Fin2W	0.088442	0.177155
Fin2DW	0.009123	0.090303
Fin3L	-0.088876	0.161731
Fin4L	0.002175	-0.001013
Fin4DW	0.025635	0.127086
TL	0.045843	0.061653
THL	0.078298	0.152418
TSL	0.018285	0.052363
Toe1L	0.106524	-0.016278
Toe2L	0.064009	0.115209
Toe3L	0.040802	0.128660
Toe4L	-0.026879	0.146104
Toe4W	-0.035289	-0.018020
Toe4DW	-0.101868	-0.035955
Toe5L	0.075004	0.135510
IMT	0.102900	0.142803
