# Supplementary material for: A new species of Leptopelis (Anura, Arthroleptidae) from the south-eastern slope of the Ethiopian Highlands, with notes on the Leptopelis gramineus species complex and the revalidation of a previously synonymised species
Source: Zookeys. 2021 Mar 11;1023:119–50. doi: 10.3897/zookeys.1023.53404 (PMC7973069; doi:10.3897/zookeys.1023.53404)
Supplement: Supplementary material 3 — Standardized Coefficients [file zookeys-1023-119-s003.pdf]

Variable	Standardized Coefficients (Leptopelis_all.sta) for Canonical Variables	
	Root 1	Root 2
HW	-0.6510	1.05261
HL	1.8456	-1.20748
ED	0.2535	-0.43949
EN	1.2569	0.39907
NS	1.2638	0.52936
SL	0.2717	-0.32776
IOD	0.5163	-0.16923
UEW	0.4381	-0.12486
IND	0.0936	-0.85470
TD	2.6920	0.10111
FLL	-1.0039	0.50148
Fin1L	-0.0105	-0.32814
Fin2L	0.5031	0.69832
Fin2W	0.7722	1.53132
Fin2DW	0.3101	-1.14461
Fin3L	-1.6056	0.38019
Fin4L	1.7442	-0.79578
Fin4DW	-1.0483	1.60361
TL	0.8474	-3.74970
THL	-0.9468	-0.72842
TSL	-1.5242	2.54850
Toe1L	0.2074	-1.65346
Toe2L	-0.4163	0.99212
Toe3L	2.3548	0.82776
Toe4L	-2.5177	-0.56075
Toe4W	0.3412	0.57476
Toe4DW	-0.1359	-0.33107
Toe5L	1.1356	-0.67551
IMT	-0.6921	-0.06712
Eigenval	121.1287	16.22248
Cum.Prop	0.8819	1.00000
