# Supplementary figures and images for: A new species of Leptopelis (Anura, Arthroleptidae) from the south-eastern slope of the Ethiopian Highlands, with notes on the Leptopelis gramineus species complex and the revalidation of a previously synonymised species
Source: Zookeys. 2021 Mar 11;1023:119–50. doi: 10.3897/zookeys.1023.53404 (PMC7973069; doi:10.3897/zookeys.1023.53404)

Mean Plot of Toe5L grouped by sp  
Leptopelis\_all.sta 256v\*47c

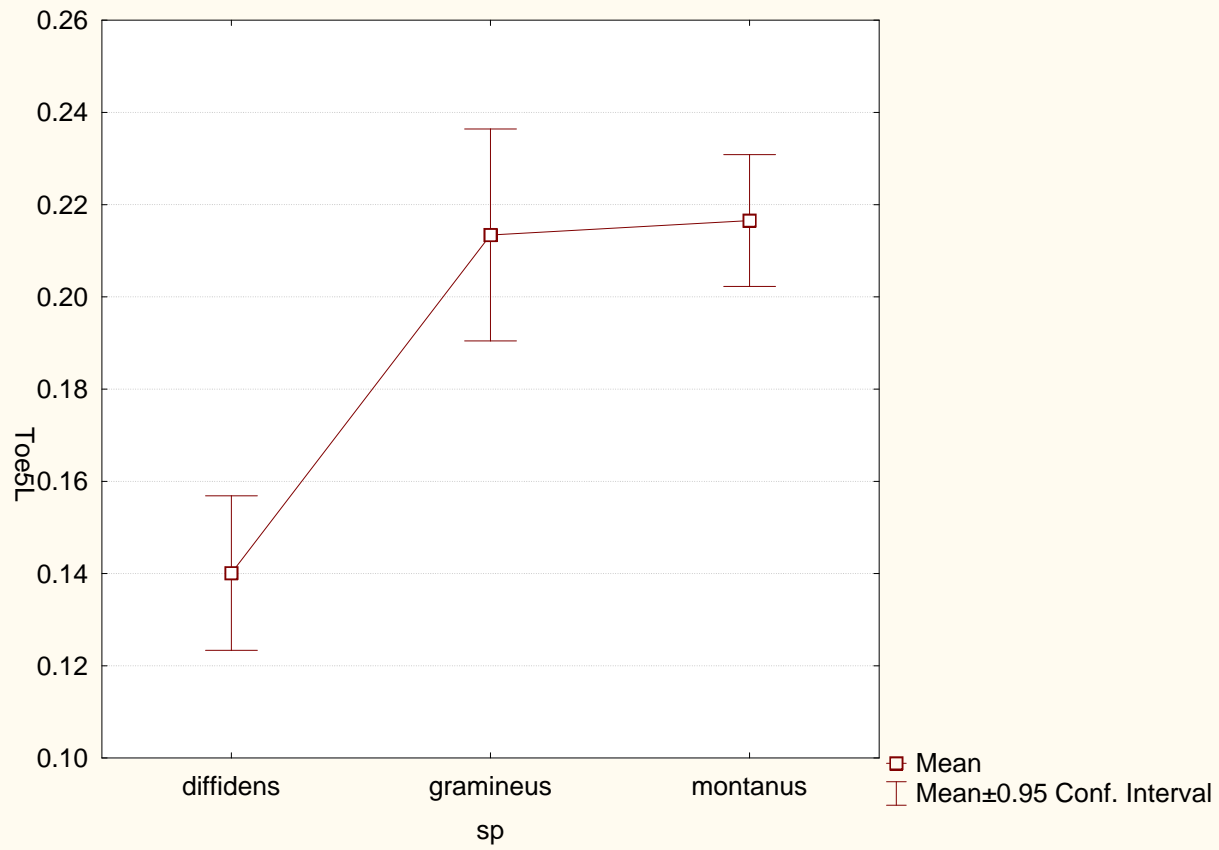

Supplement: Supplementary material 4 — Mean plots of morphometric characters grouped by species [file zookeys-1023-119-s004.zip › Mean Plot of Toe5L grouped by sp.pdf]

Mean Plot of THL grouped by sp  
Leptopelis\_all.sta 256v\*47c

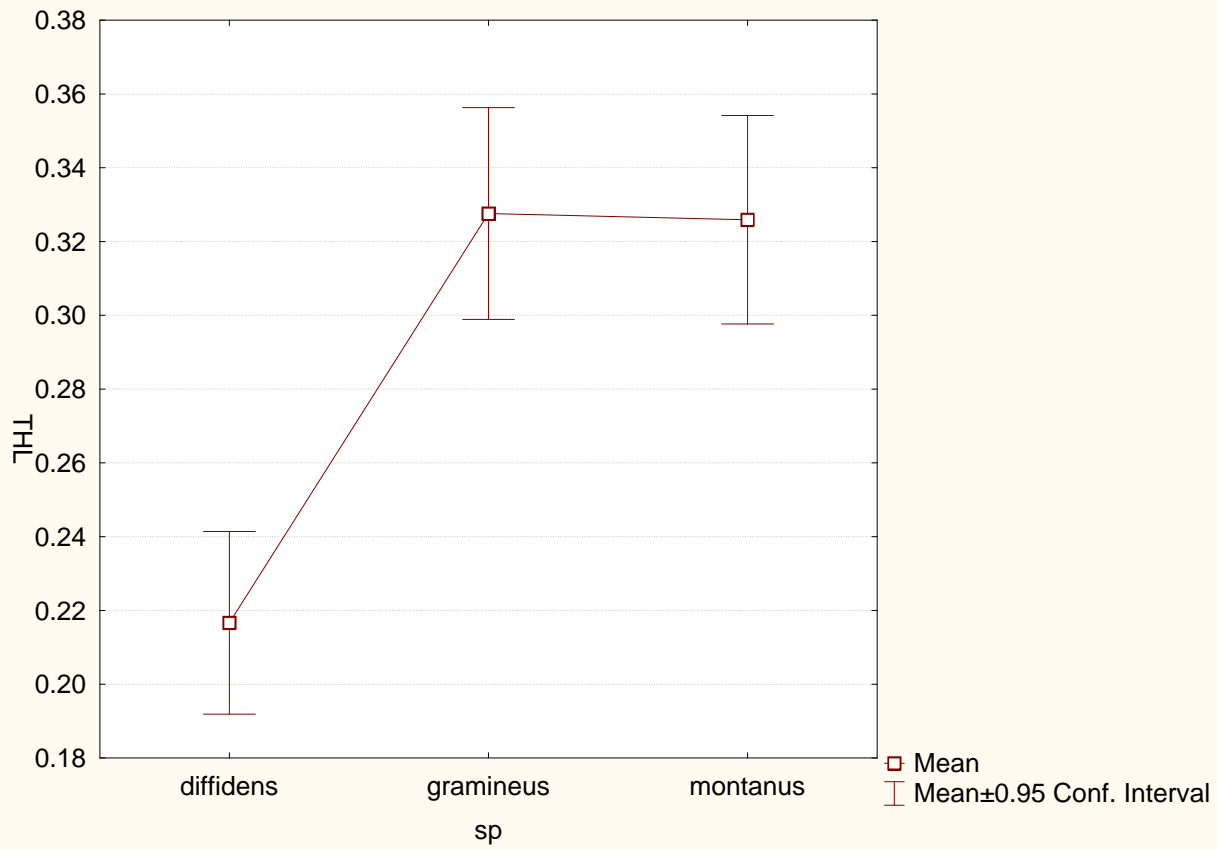

Supplement: Supplementary material 4 — Mean plots of morphometric characters grouped by species [file zookeys-1023-119-s004.zip › Mean Plot of THL grouped by sp.pdf]

Mean Plot of TSL grouped by sp  
Leptopelis\_all.sta 256v\*47c

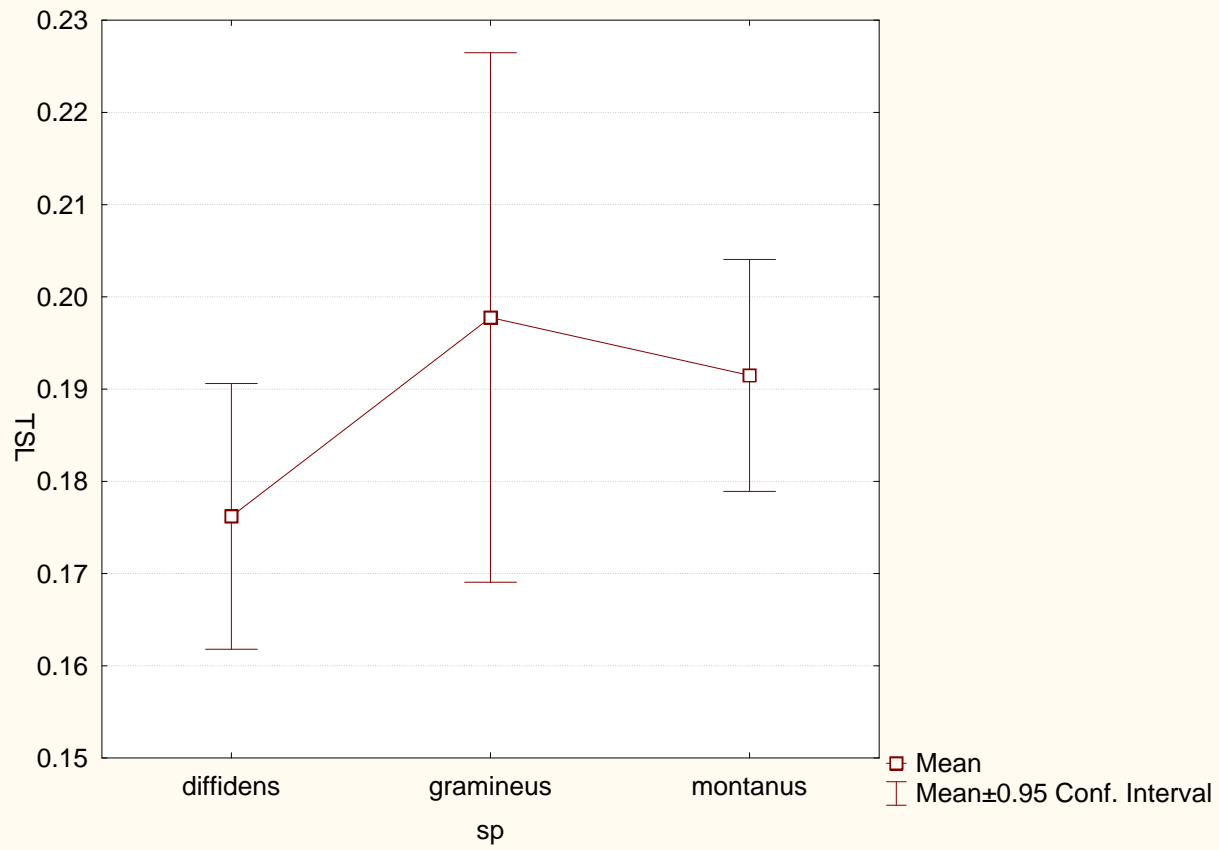

Supplement: Supplementary material 4 — Mean plots of morphometric characters grouped by species [file zookeys-1023-119-s004.zip › Mean Plot of TSL grouped by sp.pdf]

Mean Plot of Toe1L grouped by sp  
Leptopelis\_all.sta 256v\*47c

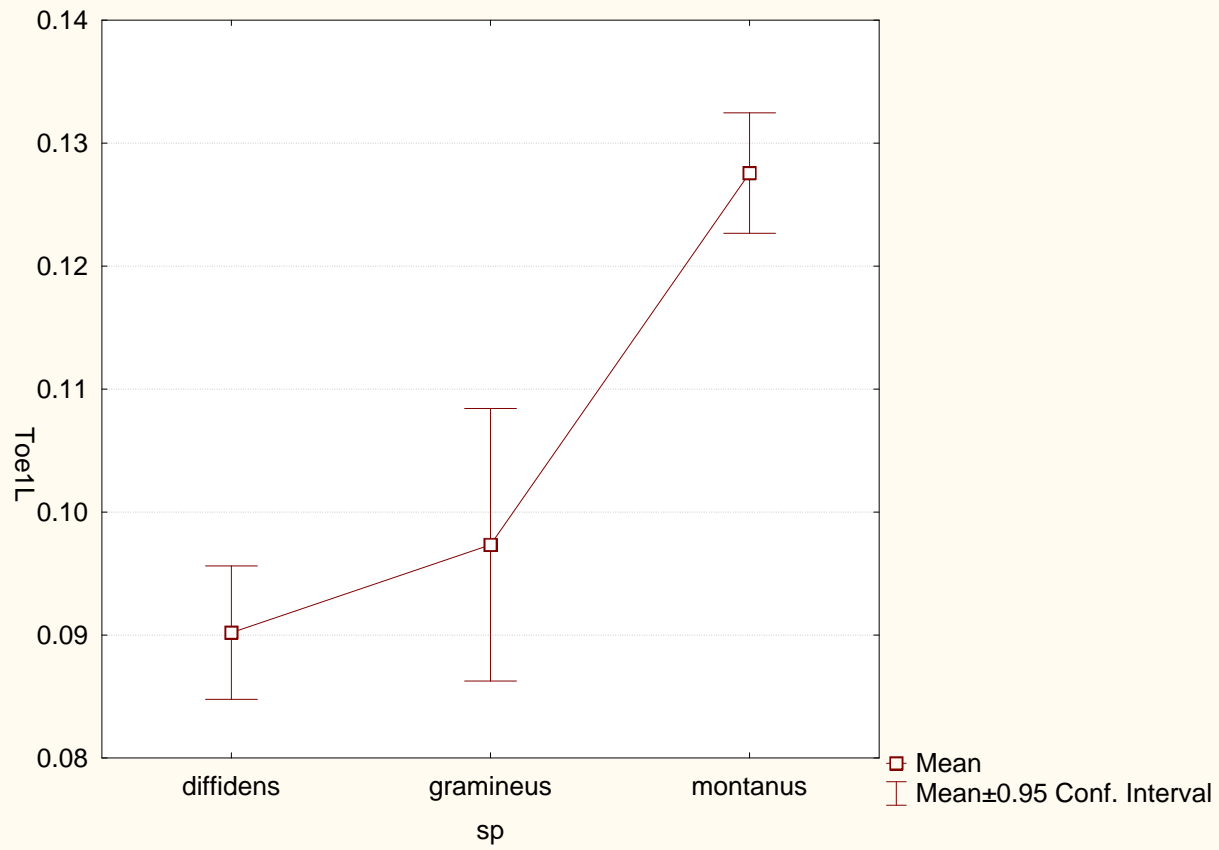

Supplement: Supplementary material 4 — Mean plots of morphometric characters grouped by species [file zookeys-1023-119-s004.zip › Mean Plot of Toe1L grouped by sp.pdf]

Mean Plot of Toe2L grouped by sp  
Leptopelis\_all.sta 256v\*47c

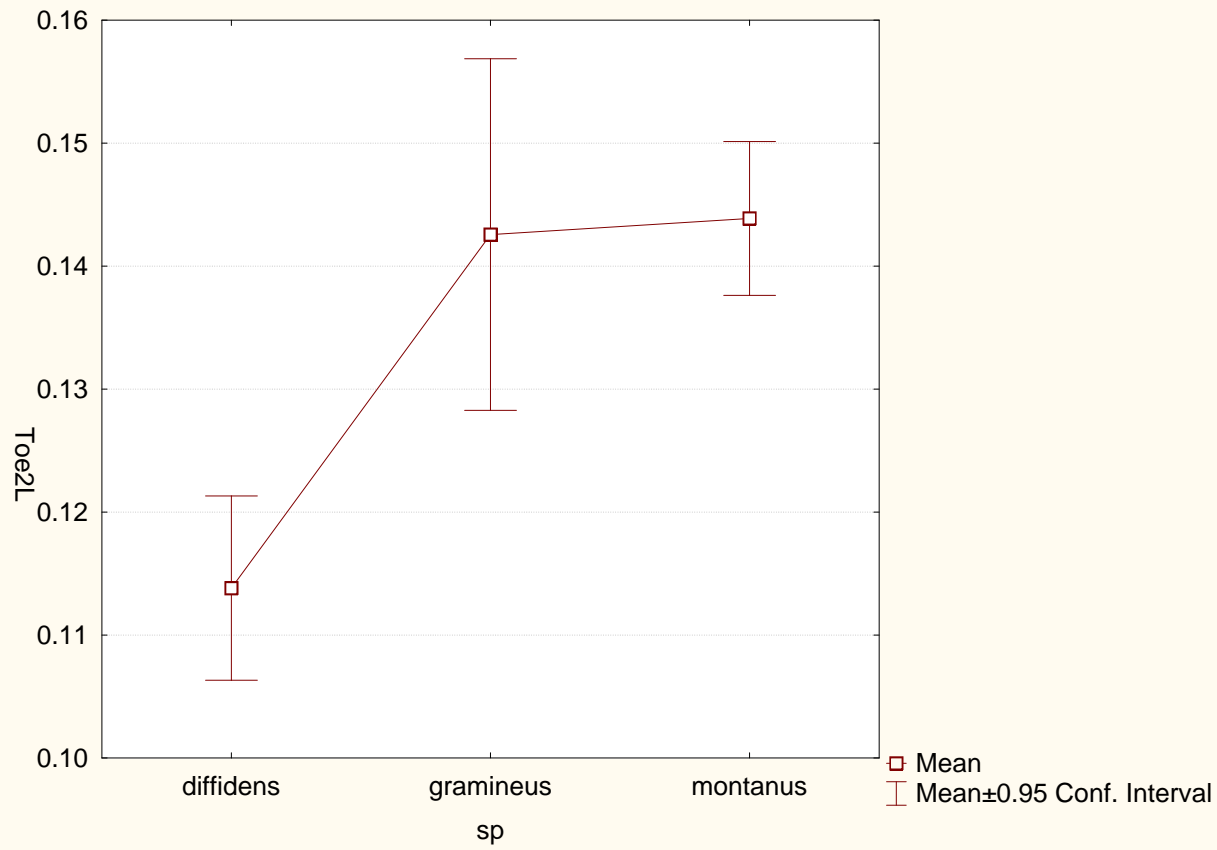

Supplement: Supplementary material 4 — Mean plots of morphometric characters grouped by species [file zookeys-1023-119-s004.zip › Mean Plot of Toe2L grouped by sp.pdf]

Mean Plot of Toe3L grouped by sp  
Leptopelis\_all.sta 256v\*47c

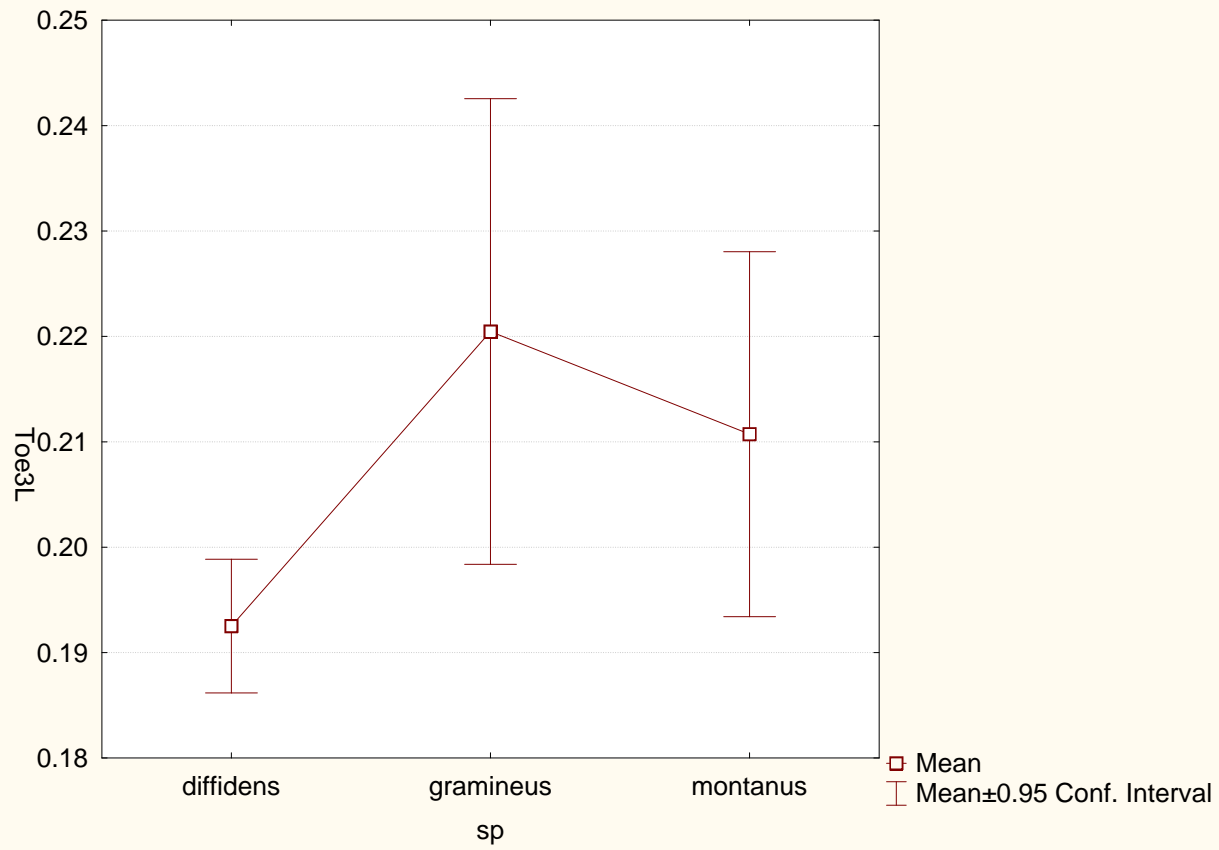

Supplement: Supplementary material 4 — Mean plots of morphometric characters grouped by species [file zookeys-1023-119-s004.zip › Mean Plot of Toe3L grouped by sp.pdf]

Mean Plot of Toe4L grouped by sp  
Leptopelis\_all.sta 256v\*47c

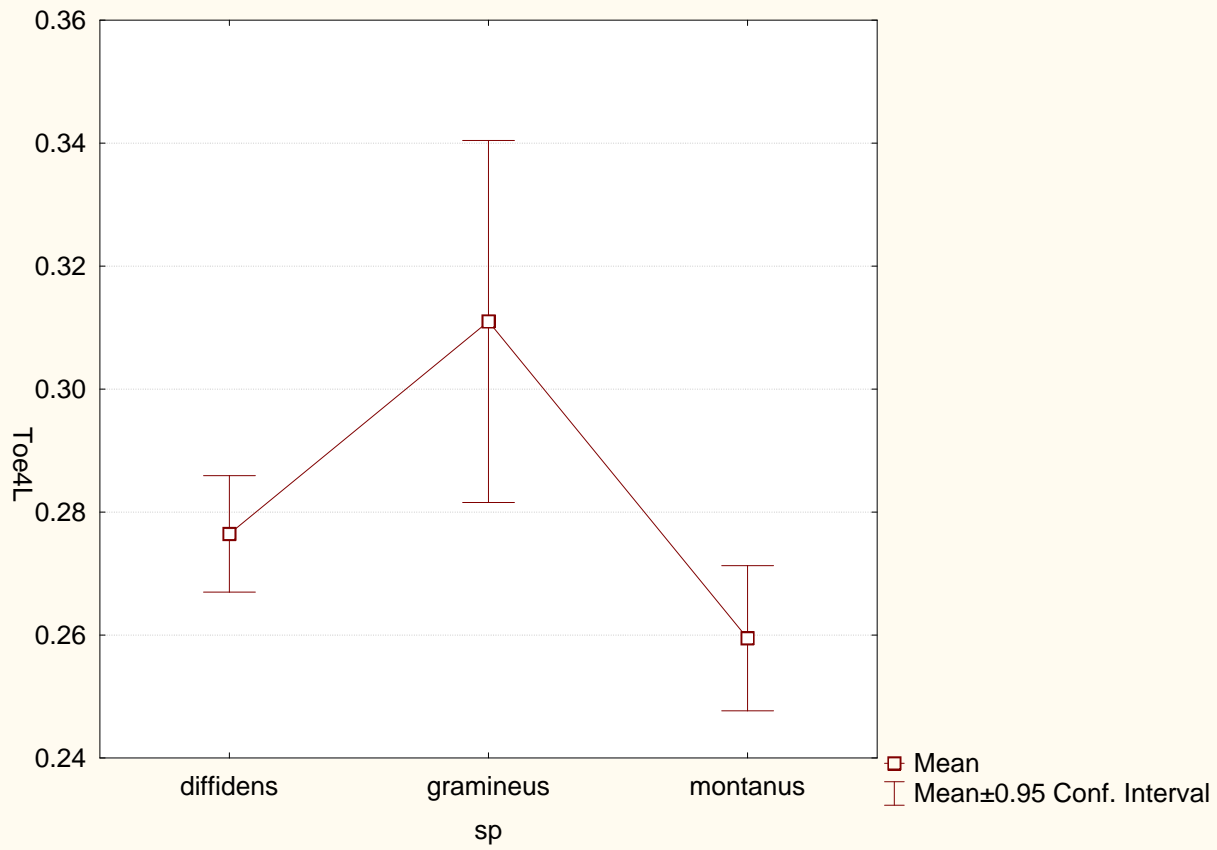

Supplement: Supplementary material 4 — Mean plots of morphometric characters grouped by species [file zookeys-1023-119-s004.zip › Mean Plot of Toe4L grouped by sp.pdf]

Mean Plot of Toe4W grouped by sp  
Leptopelis\_all.sta 256v\*47c

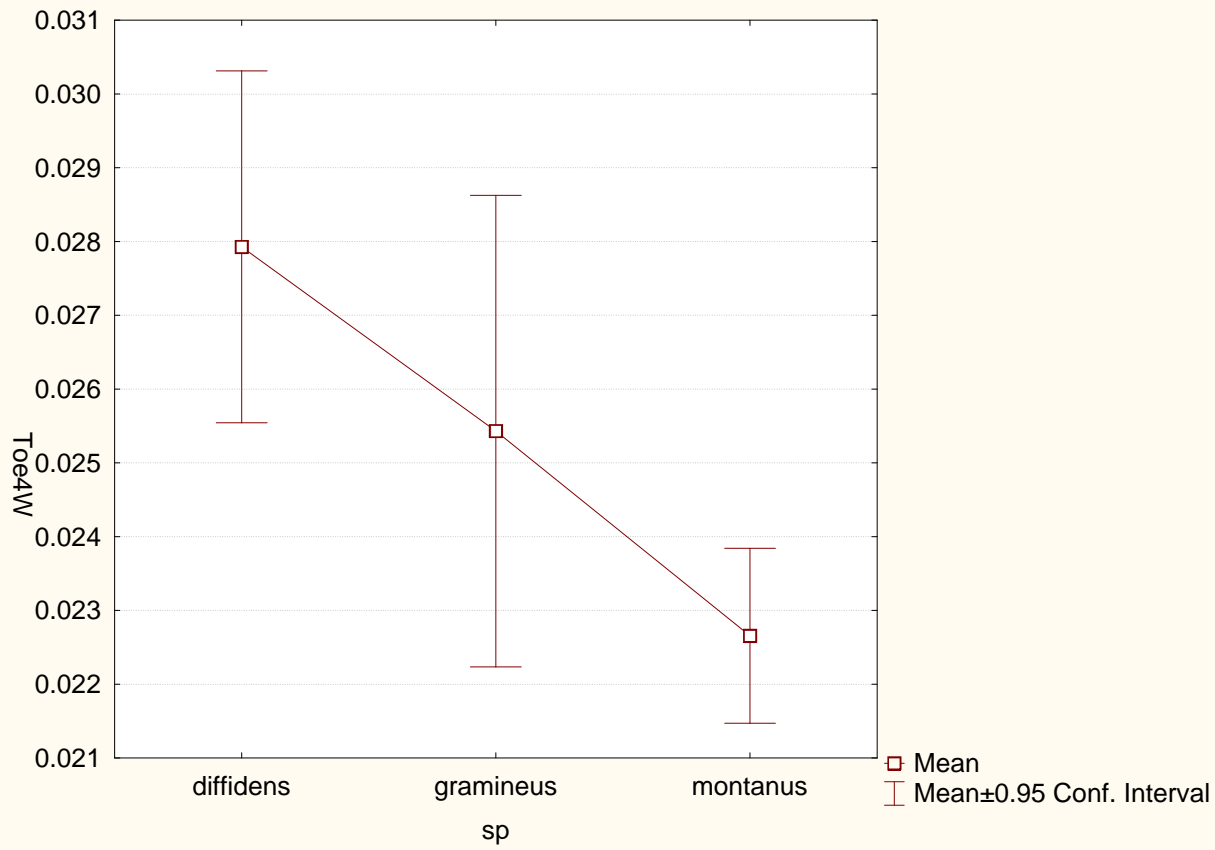

Supplement: Supplementary material 4 — Mean plots of morphometric characters grouped by species [file zookeys-1023-119-s004.zip › Mean Plot of Toe4W grouped by sp.pdf]

Mean Plot of IMT grouped by sp  
Leptopelis\_all.sta 256v\*47c

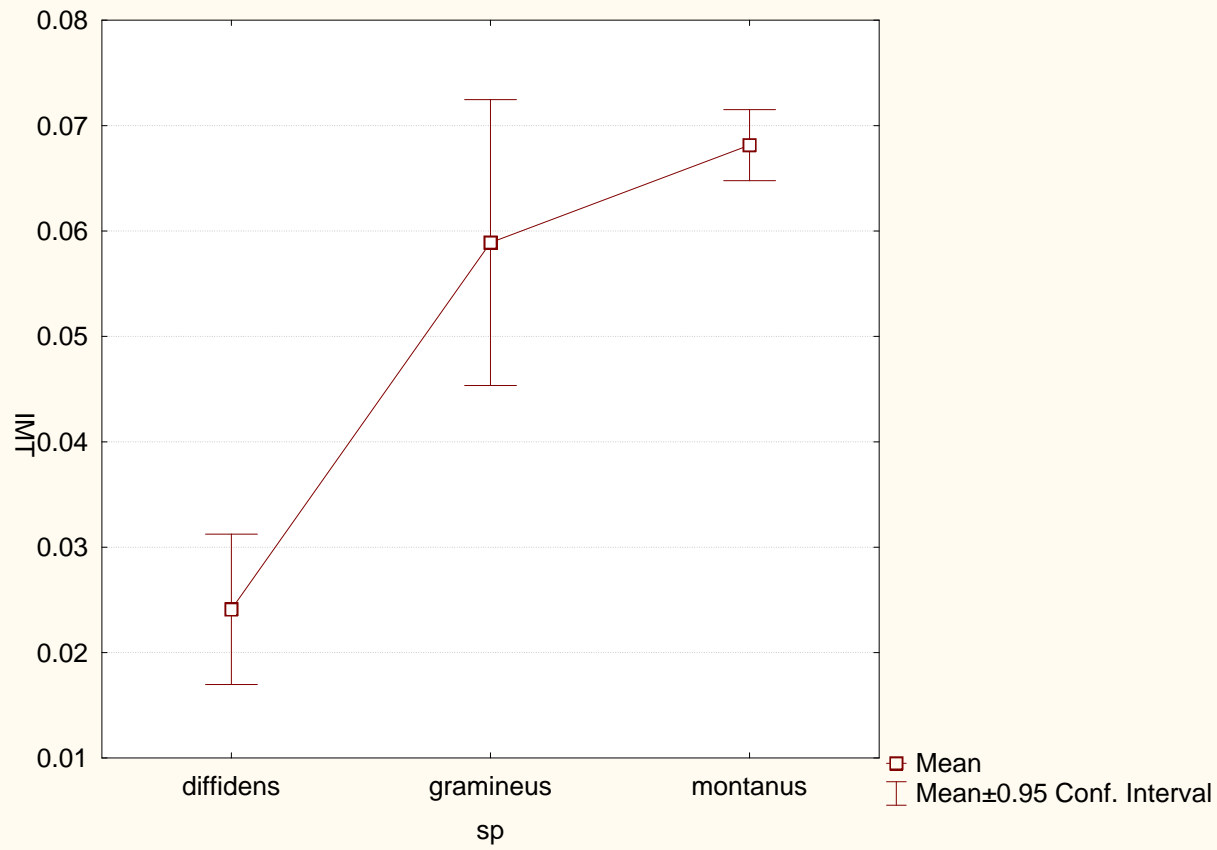

Supplement: Supplementary material 4 — Mean plots of morphometric characters grouped by species [file zookeys-1023-119-s004.zip › Mean Plot of IMT grouped by sp.pdf]

Mean Plot of IMTW grouped by sp

Leptopelis\_all.sta 256v\*47c

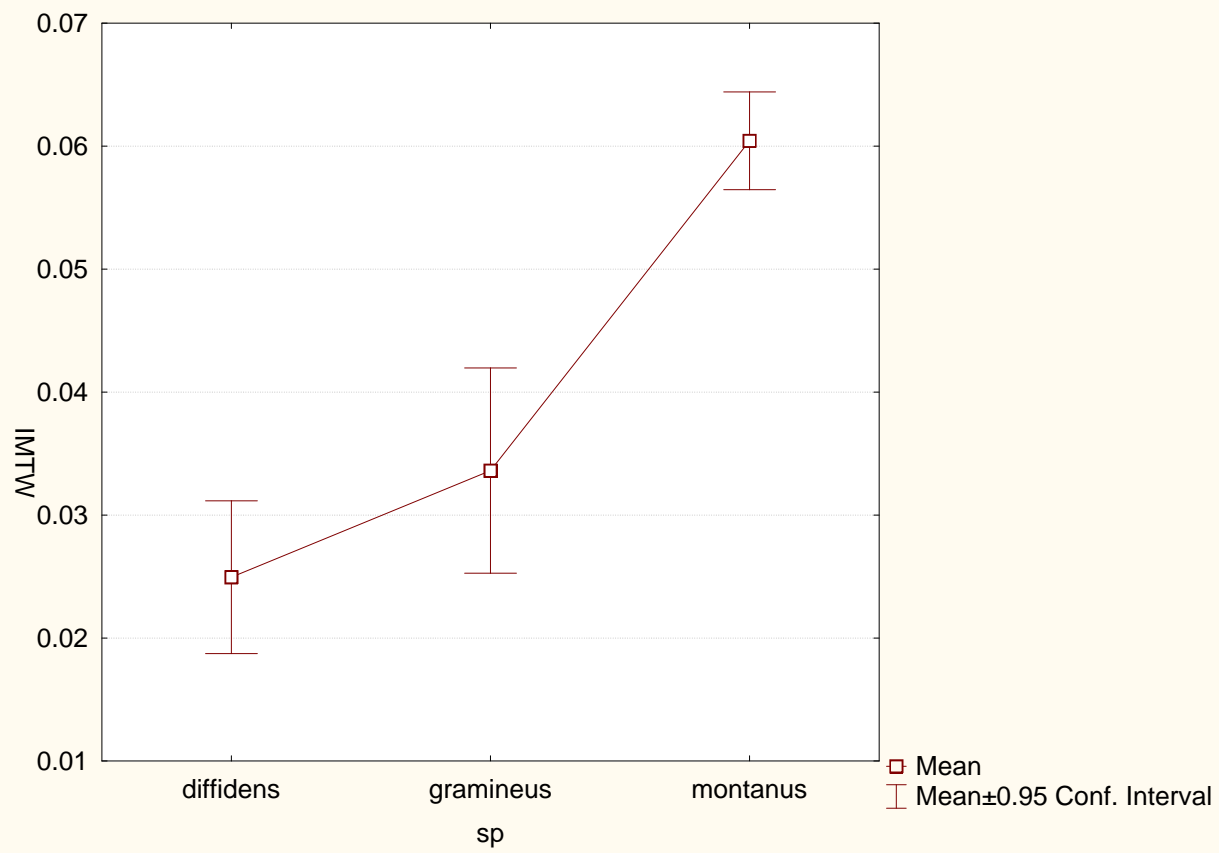

Supplement: Supplementary material 4 — Mean plots of morphometric characters grouped by species [file zookeys-1023-119-s004.zip › Mean Plot of IMTW grouped by sp.pdf]

Mean Plot of FLL grouped by sp  
Leptopelis\_all.sta 256v\*47c

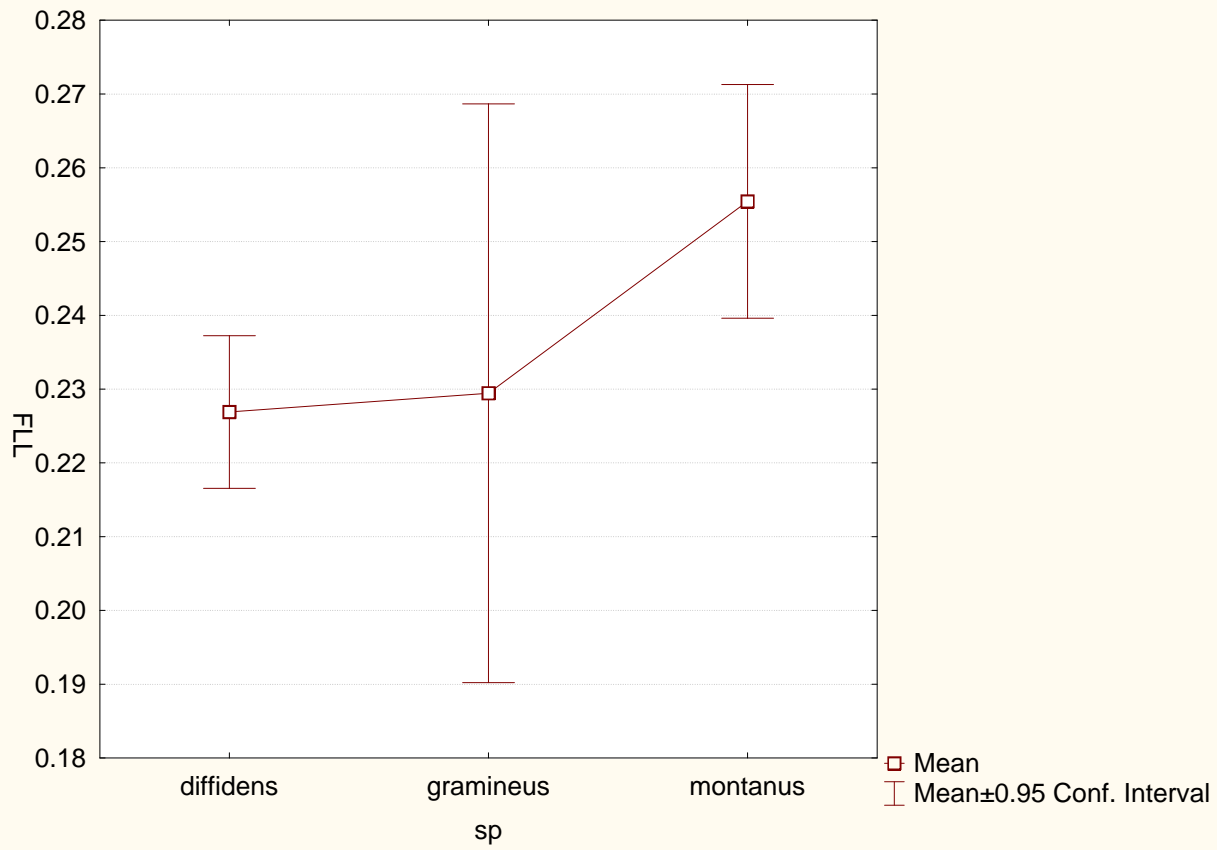

Supplement: Supplementary material 4 — Mean plots of morphometric characters grouped by species [file zookeys-1023-119-s004.zip › Mean Plot of FLL grouped by sp.pdf]

Mean Plot of Fin1L grouped by sp  
Leptopelis\_all.sta 256v\*47c

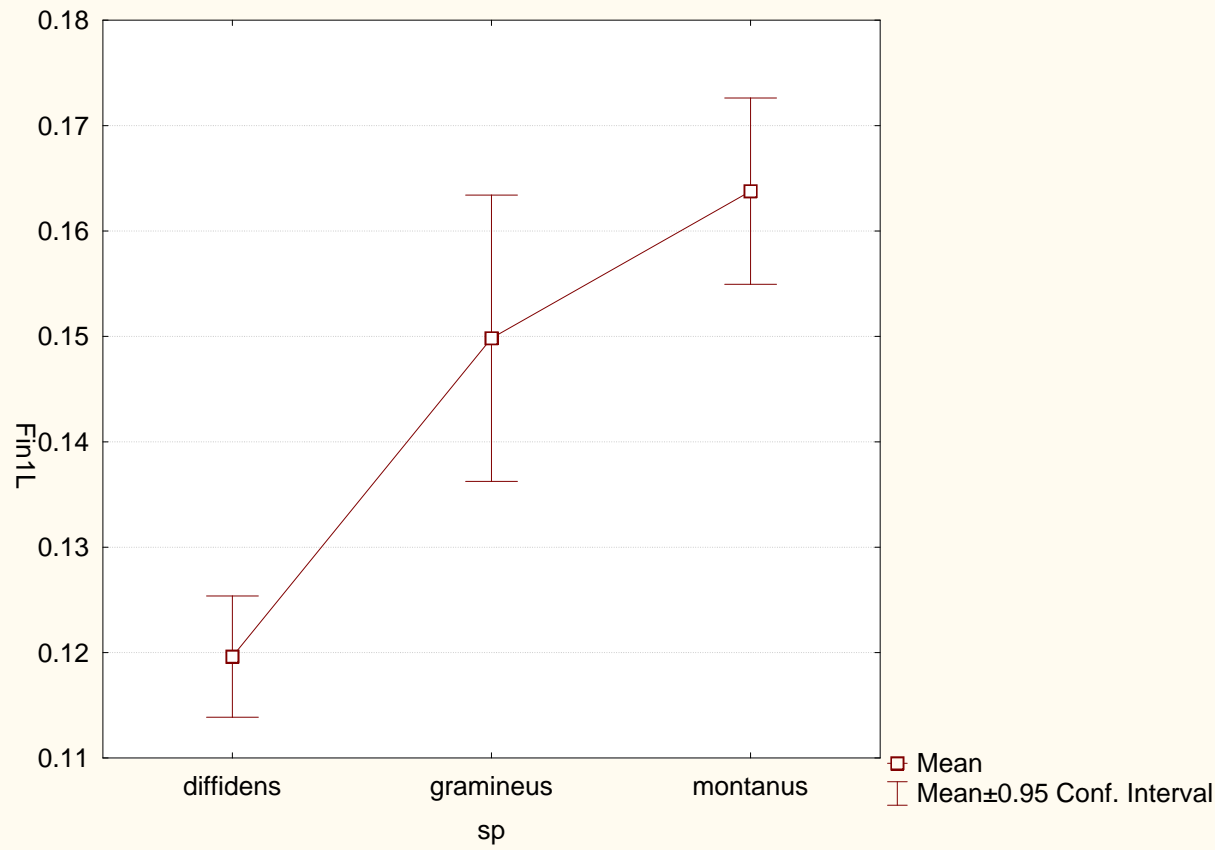

Supplement: Supplementary material 4 — Mean plots of morphometric characters grouped by species [file zookeys-1023-119-s004.zip › Mean Plot of Fin1L grouped by sp.pdf]

Mean Plot of Fin2L grouped by sp  
Leptopelis\_all.sta 256v\*47c

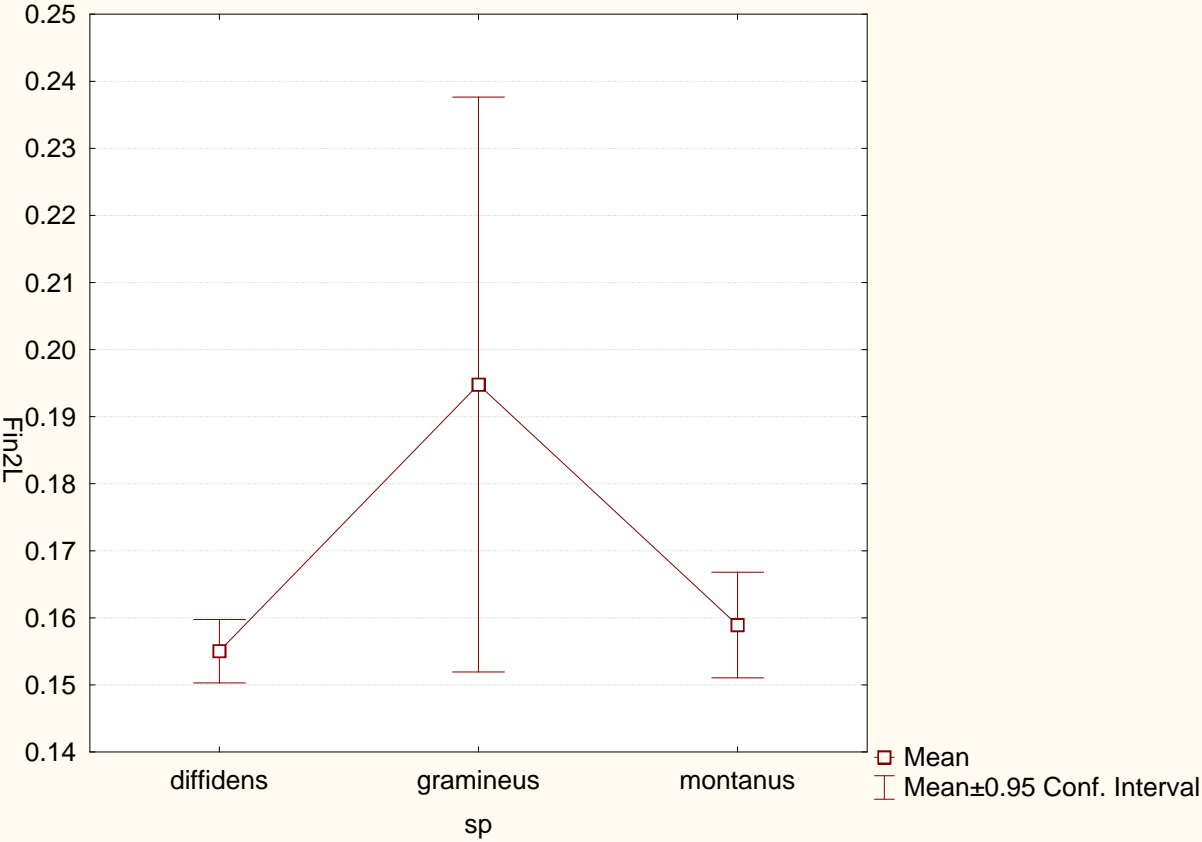

Supplement: Supplementary material 4 — Mean plots of morphometric characters grouped by species [file zookeys-1023-119-s004.zip › Mean Plot of Fin2L grouped by sp.pdf]

Mean Plot of Fin2W grouped by sp  
Leptopelis\_all.sta 256v\*47c

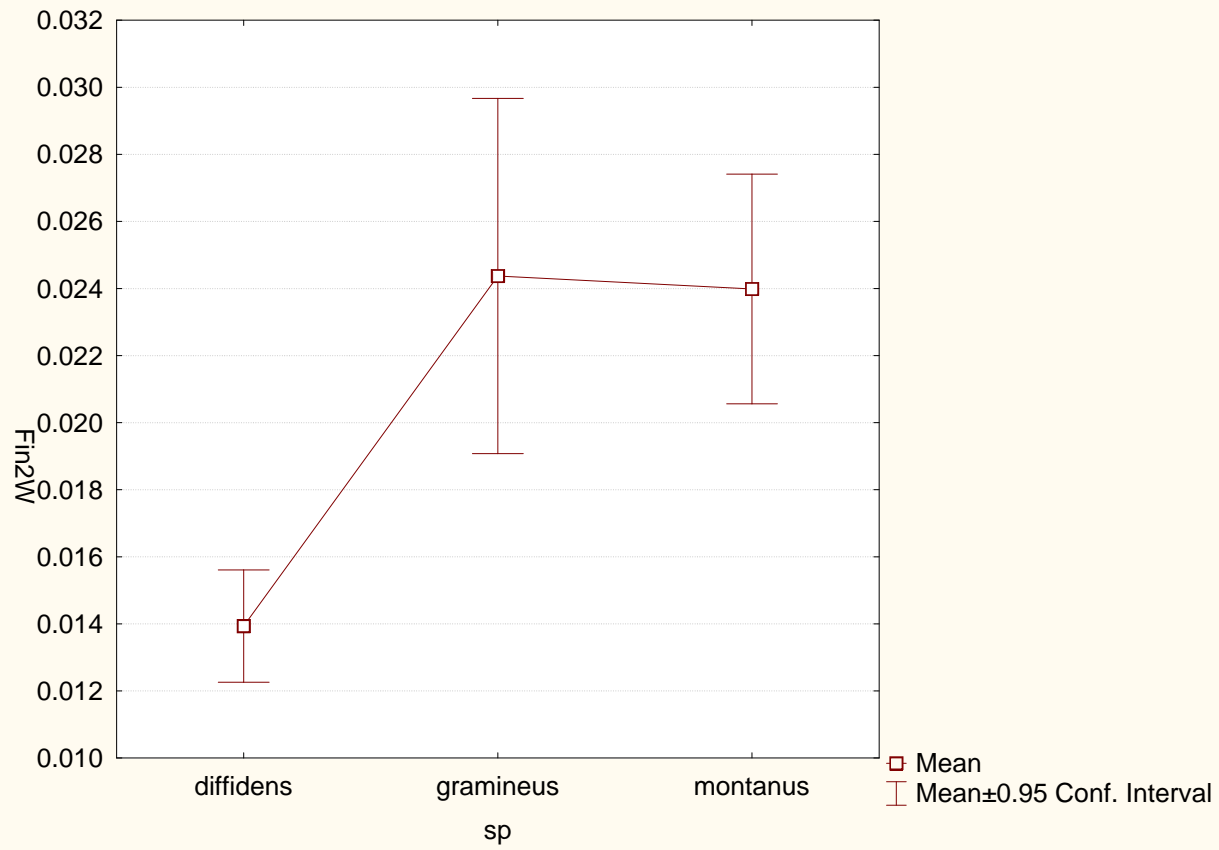

Supplement: Supplementary material 4 — Mean plots of morphometric characters grouped by species [file zookeys-1023-119-s004.zip › Mean Plot of Fin2W grouped by sp.pdf]

Mean Plot of Fin2DW grouped by sp  
Leptopelis\_all.sta 256v\*47c

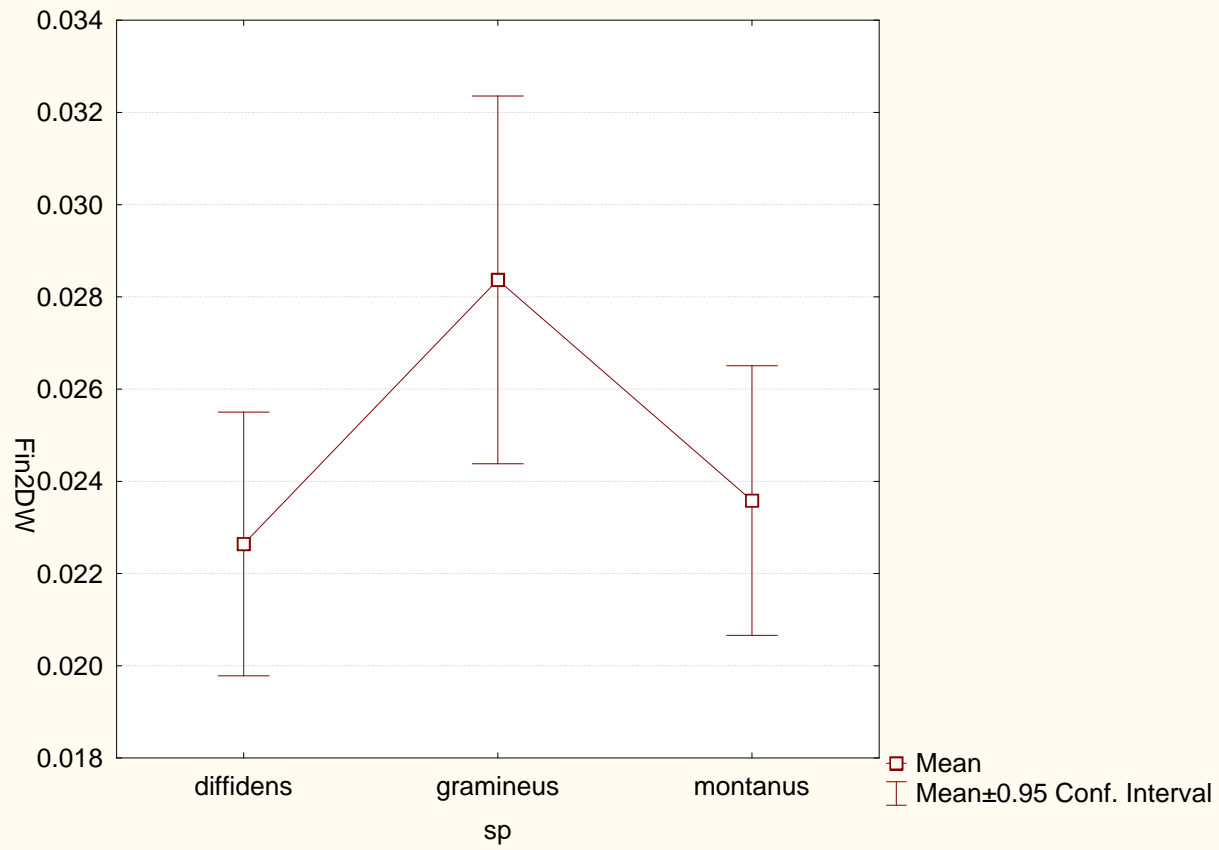

Supplement: Supplementary material 4 — Mean plots of morphometric characters grouped by species [file zookeys-1023-119-s004.zip › Mean Plot of Fin2DW grouped by sp.pdf]

Mean Plot of Fin3L grouped by sp  
Leptopelis\_all.sta 256v\*47c

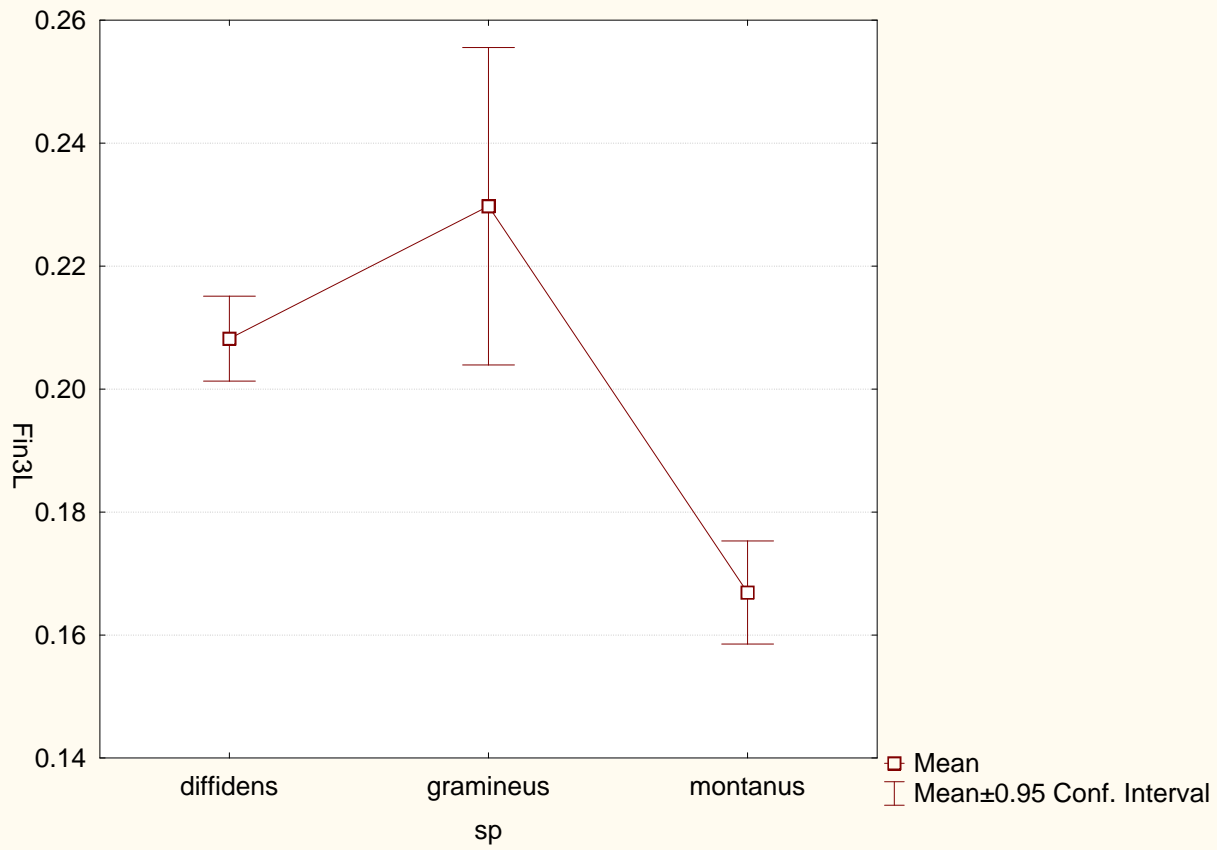

Supplement: Supplementary material 4 — Mean plots of morphometric characters grouped by species [file zookeys-1023-119-s004.zip › Mean Plot of Fin3L grouped by sp.pdf]

Mean Plot of Fin4L grouped by sp  
Leptopelis\_all.sta 256v\*47c

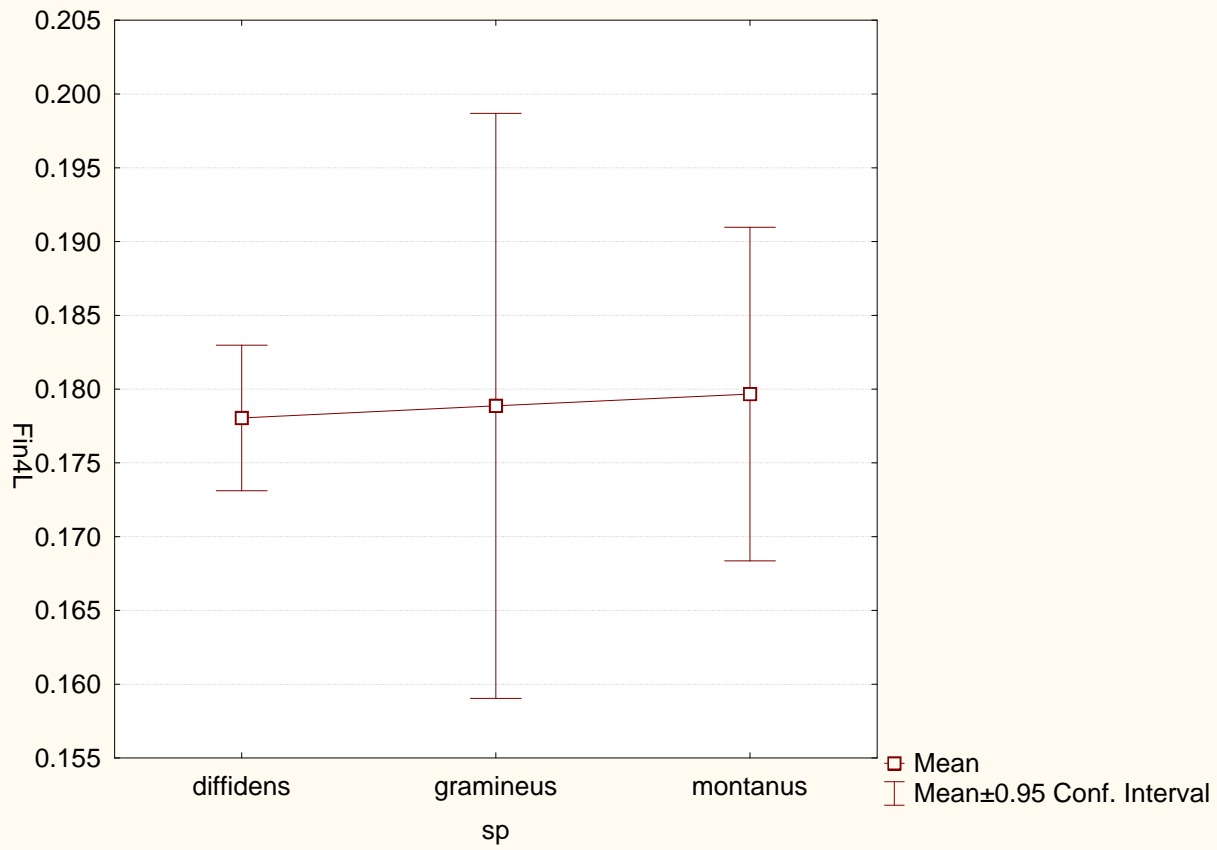

Supplement: Supplementary material 4 — Mean plots of morphometric characters grouped by species [file zookeys-1023-119-s004.zip › Mean Plot of Fin4L grouped by sp.pdf]

Mean Plot of Fin4W grouped by sp  
Leptopelis\_all.sta 256v\*47c

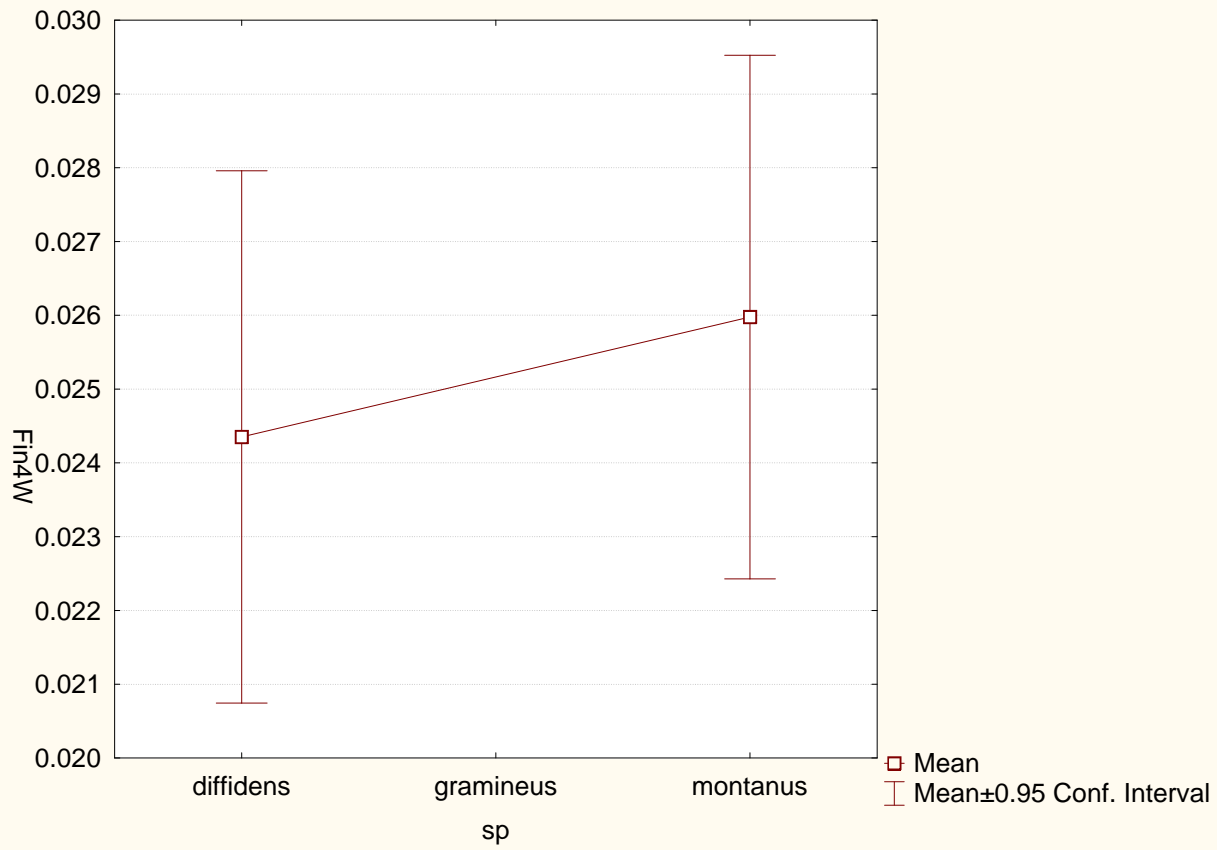

Supplement: Supplementary material 4 — Mean plots of morphometric characters grouped by species [file zookeys-1023-119-s004.zip › Mean Plot of Fin4W grouped by sp.pdf]

Mean Plot of Fin4DW grouped by sp  
Leptopelis\_all.sta 256v\*47c

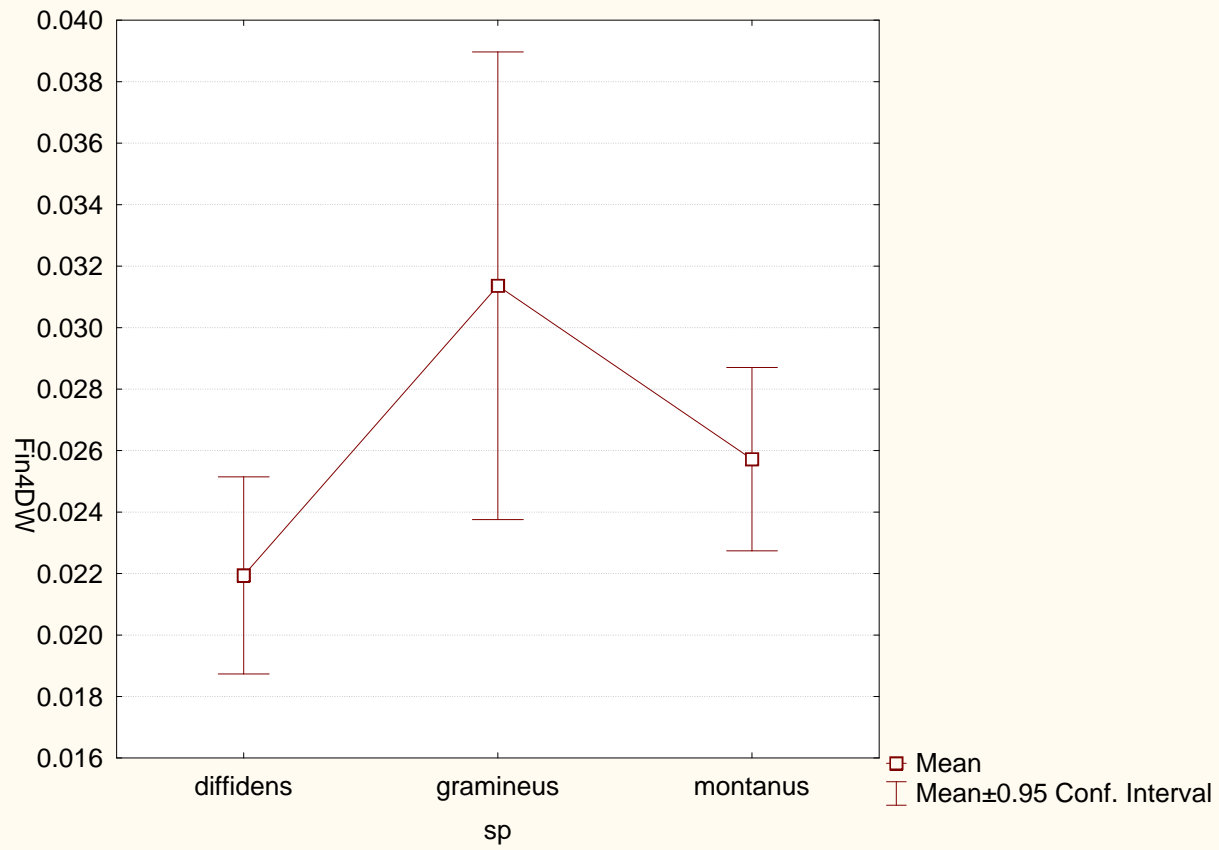

Supplement: Supplementary material 4 — Mean plots of morphometric characters grouped by species [file zookeys-1023-119-s004.zip › Mean Plot of Fin4DW grouped by sp.pdf]

Mean Plot of TL grouped by sp  
Leptopelis\_all.sta 256v\*47c

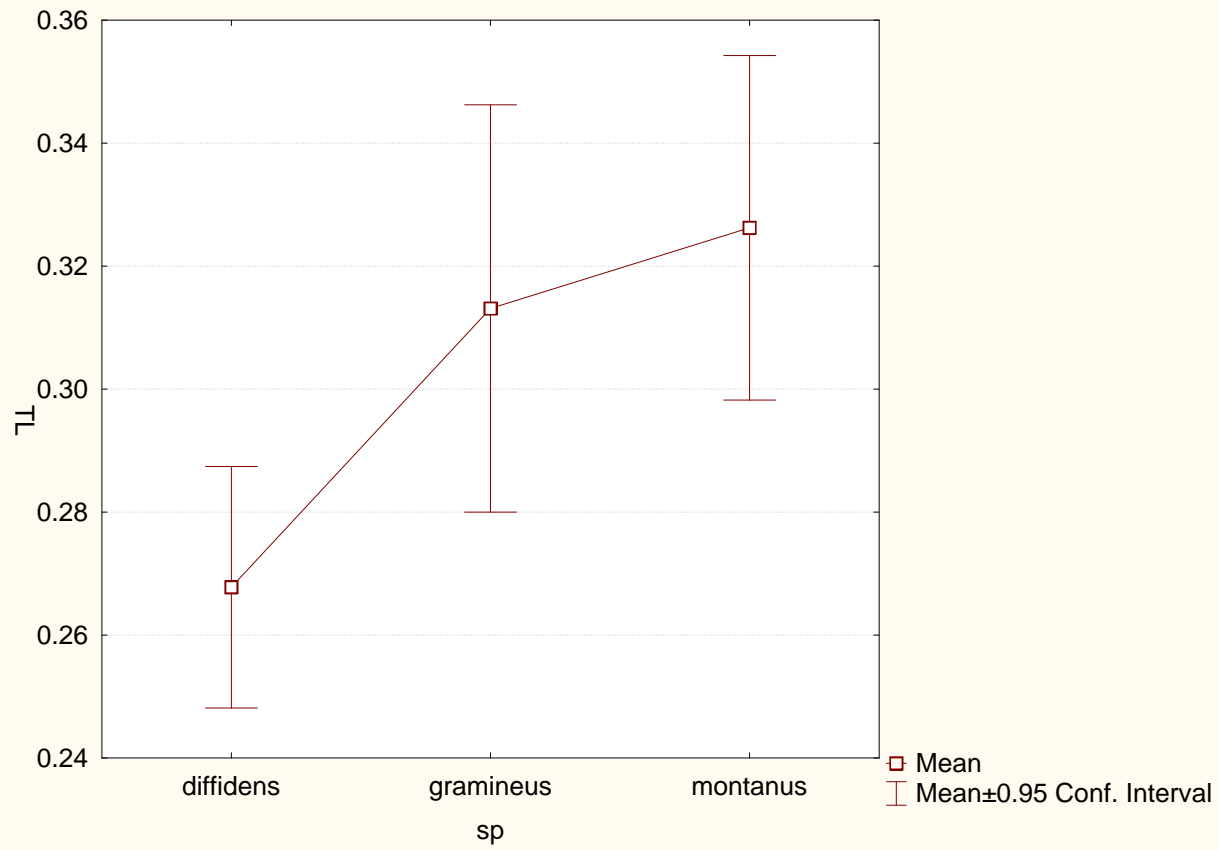

Supplement: Supplementary material 4 — Mean plots of morphometric characters grouped by species [file zookeys-1023-119-s004.zip › Mean Plot of TL grouped by sp.pdf]

Mean Plot of HW grouped by sp  
Leptopelis\_all.sta 256v\*47c

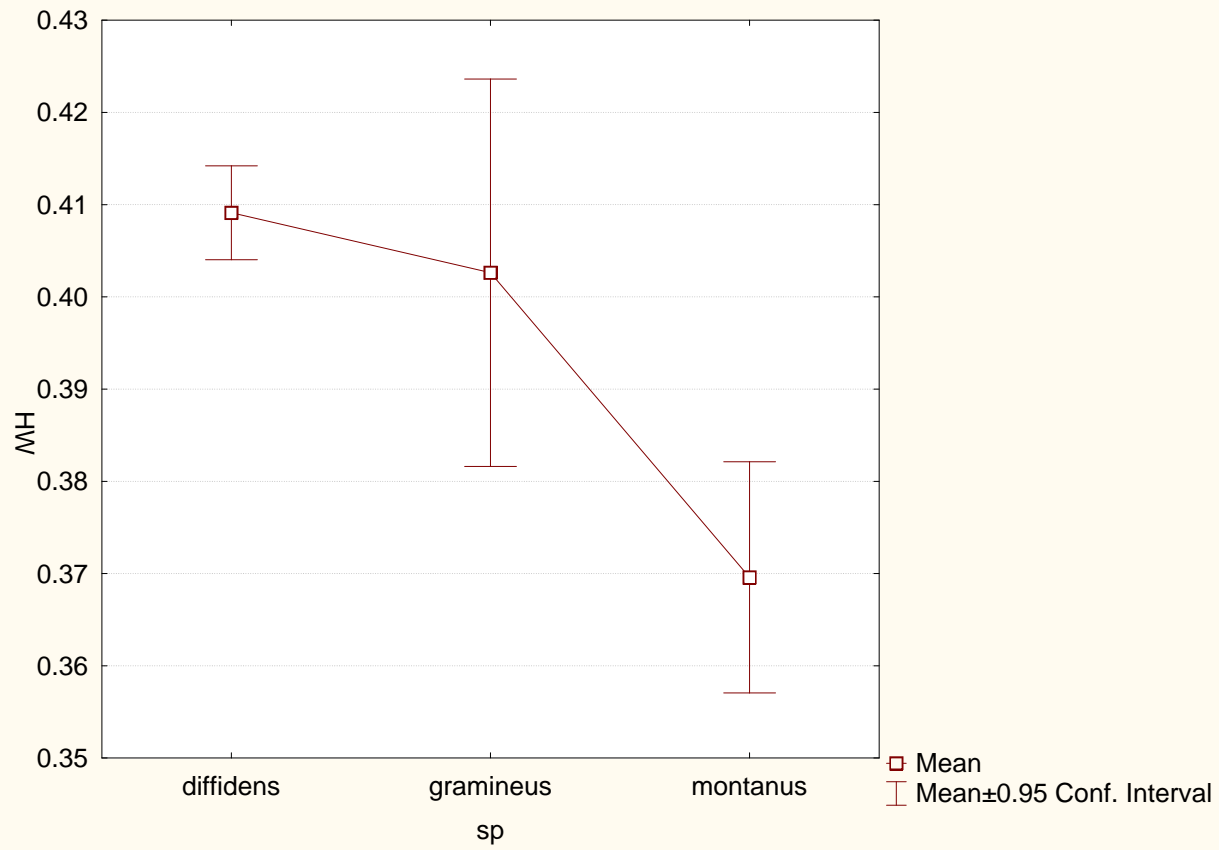

Supplement: Supplementary material 4 — Mean plots of morphometric characters grouped by species [file zookeys-1023-119-s004.zip › Mean Plot of HW grouped by sp.pdf]

Mean Plot of HL grouped by sp  
Leptopelis\_all.sta 256v\*47c

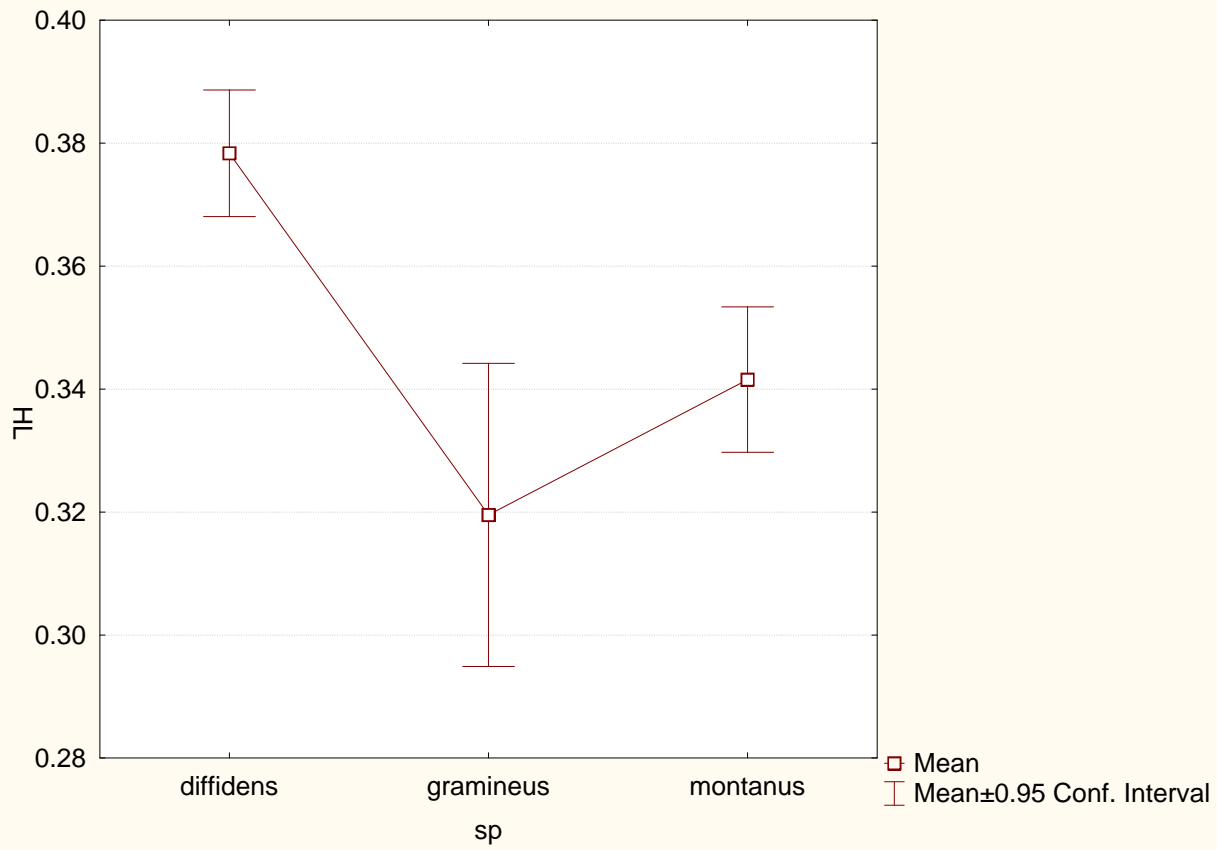

Supplement: Supplementary material 4 — Mean plots of morphometric characters grouped by species [file zookeys-1023-119-s004.zip › Mean Plot of HL grouped by sp.pdf]

Mean Plot of ED grouped by sp  
Leptopelis\_all.sta 256v\*47c

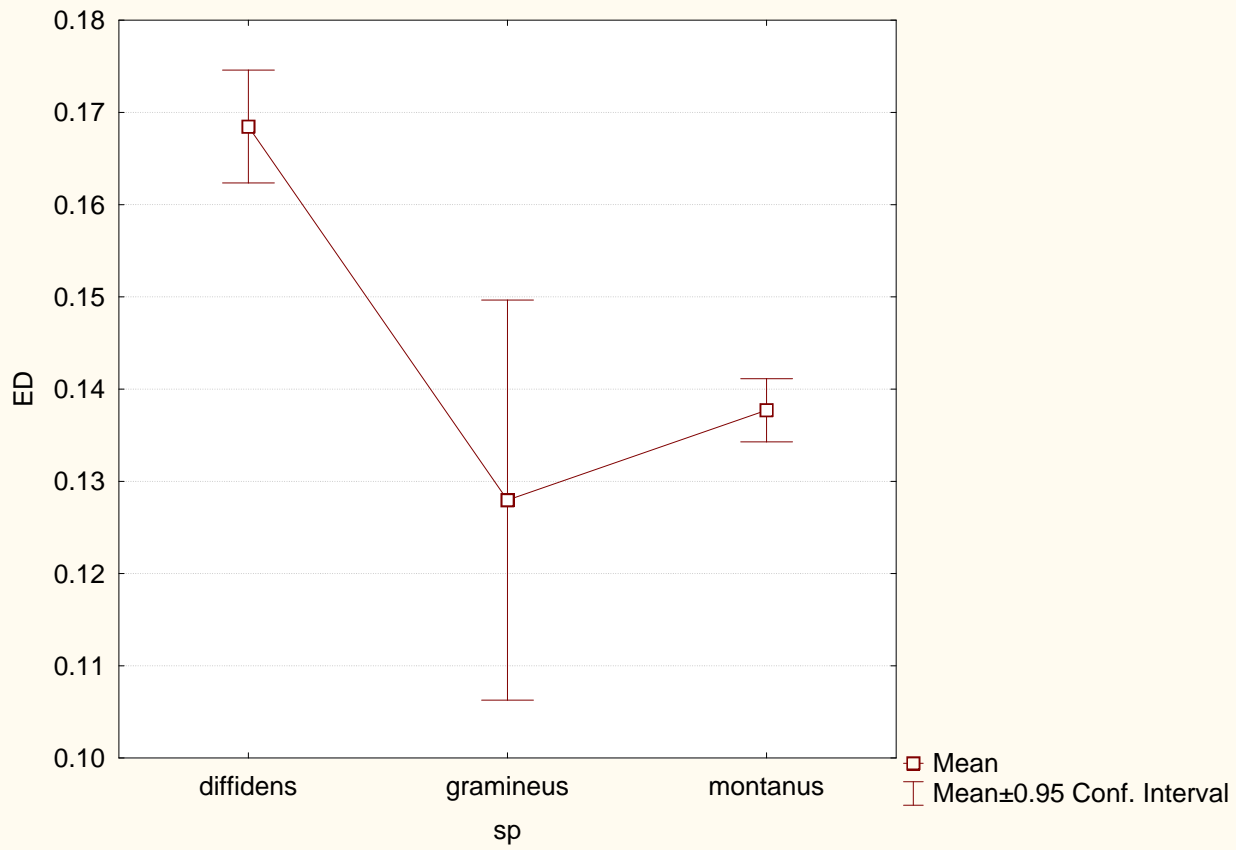

Supplement: Supplementary material 4 — Mean plots of morphometric characters grouped by species [file zookeys-1023-119-s004.zip › Mean Plot of ED grouped by sp.pdf]

Mean Plot of EN grouped by sp  
Leptopelis\_all.sta 256v\*47c

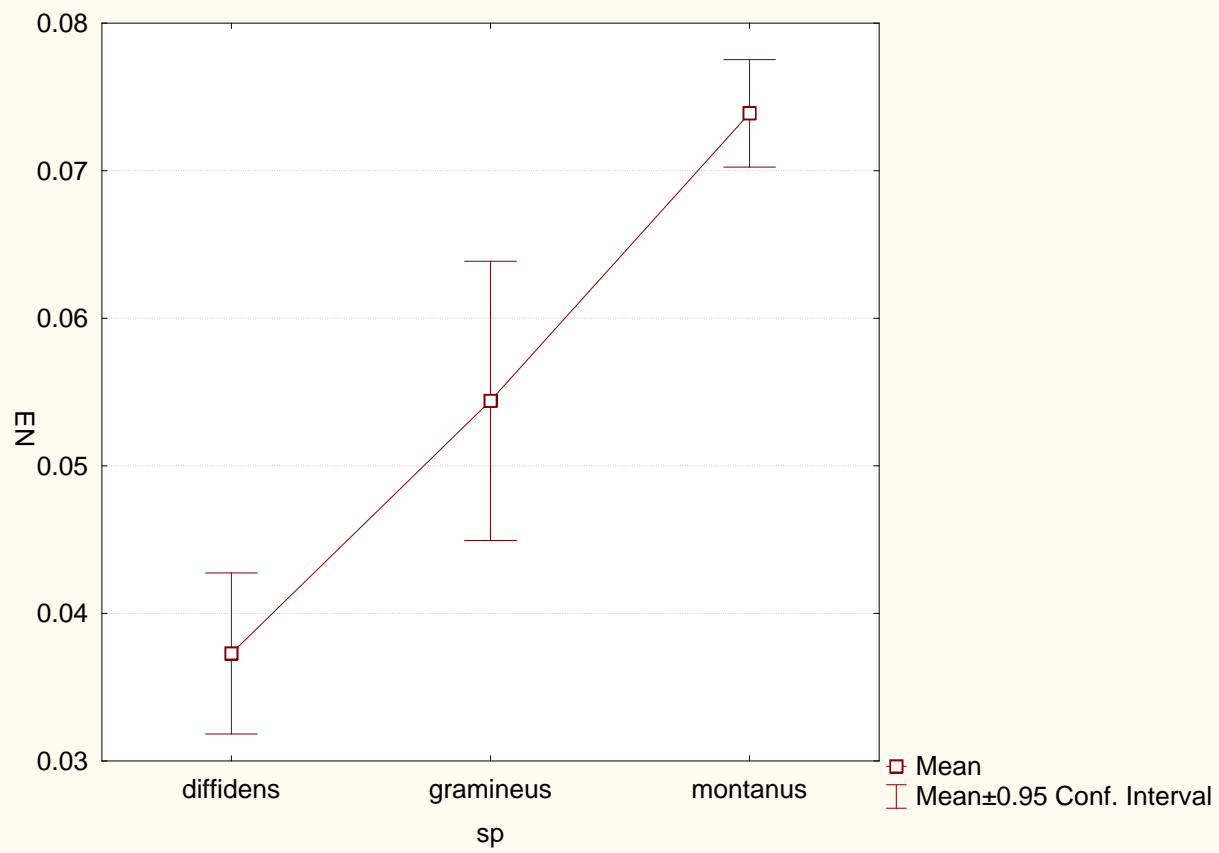

Supplement: Supplementary material 4 — Mean plots of morphometric characters grouped by species [file zookeys-1023-119-s004.zip › Mean Plot of EN grouped by sp.pdf]

Mean Plot of ETD grouped by sp  
Leptopelis\_all.sta 256v\*47c

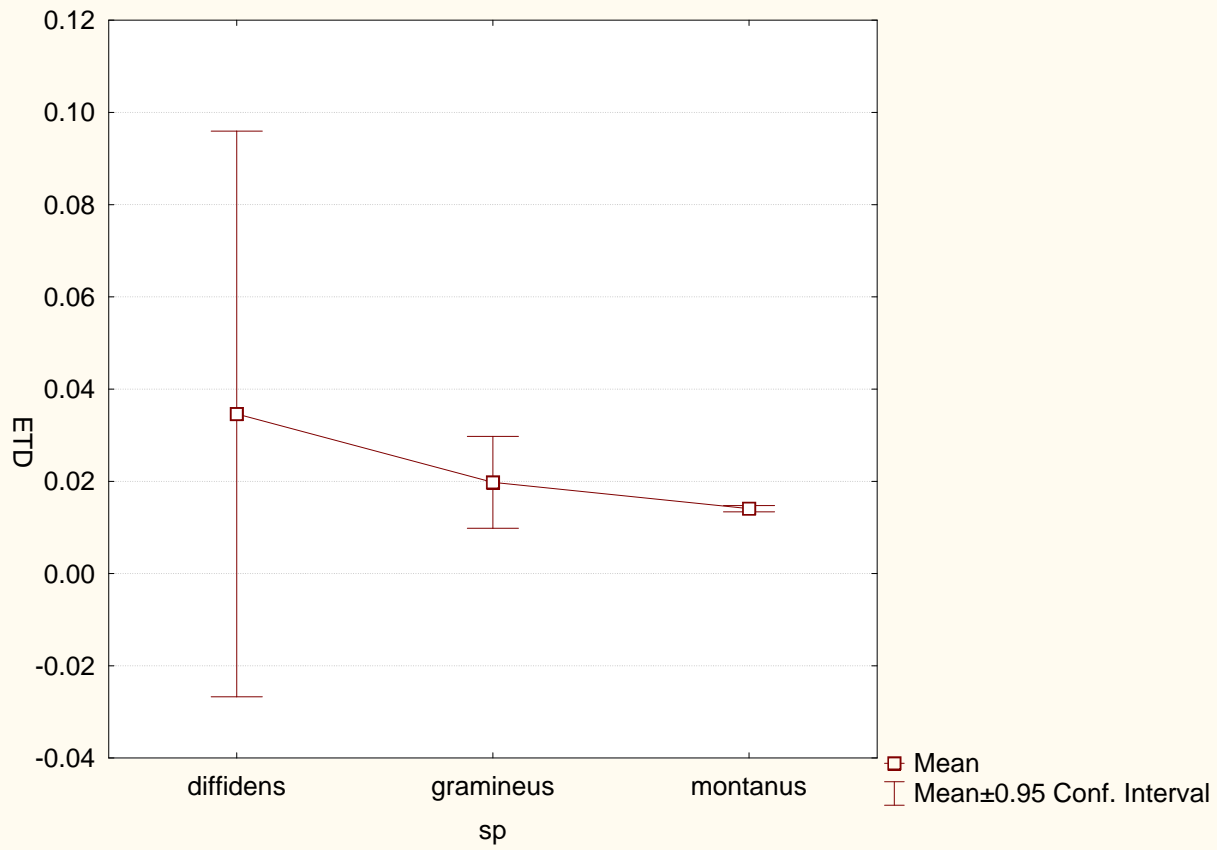

Supplement: Supplementary material 4 — Mean plots of morphometric characters grouped by species [file zookeys-1023-119-s004.zip › Mean Plot of ETD grouped by sp.pdf]

Mean Plot of Toe4DW grouped by sp  
Leptopelis\_all.sta 256v\*47c

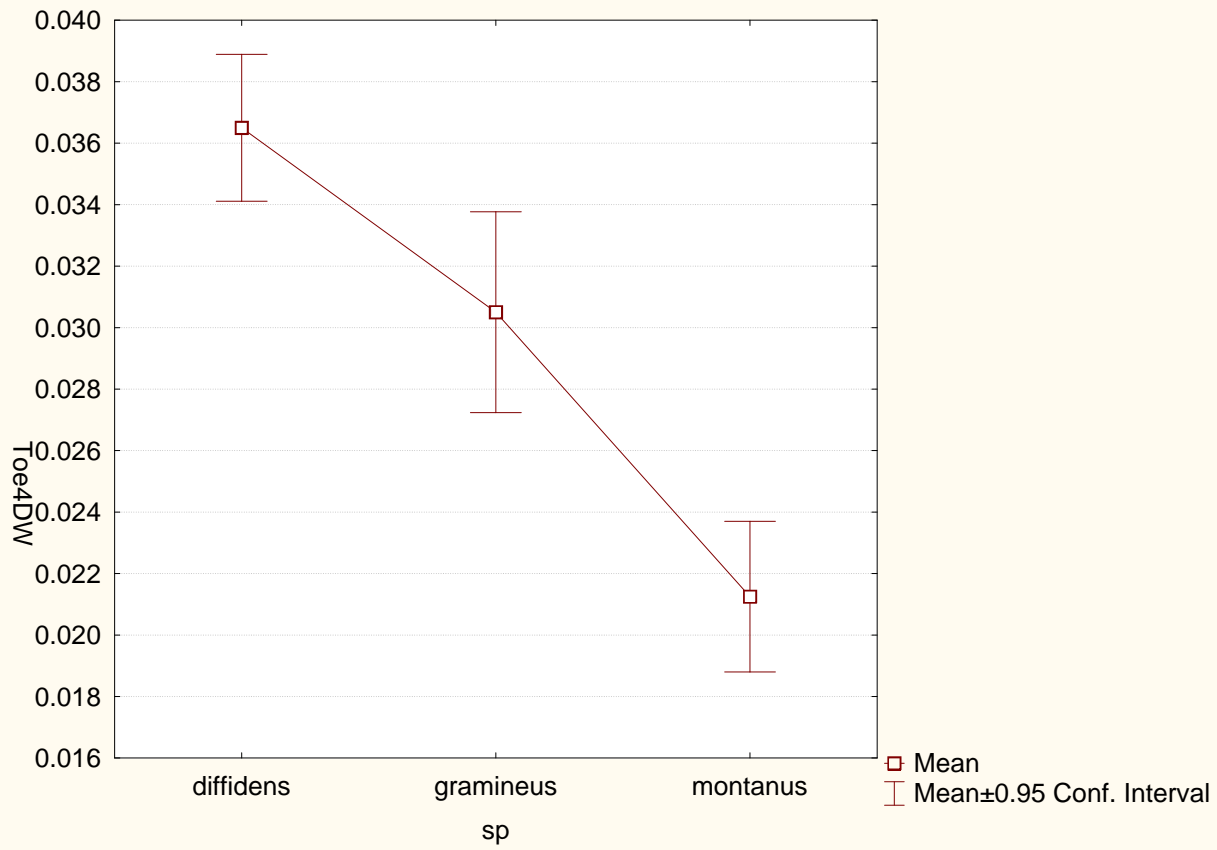

Supplement: Supplementary material 4 — Mean plots of morphometric characters grouped by species [file zookeys-1023-119-s004.zip › Mean Plot of Toe4DW grouped by sp.pdf]

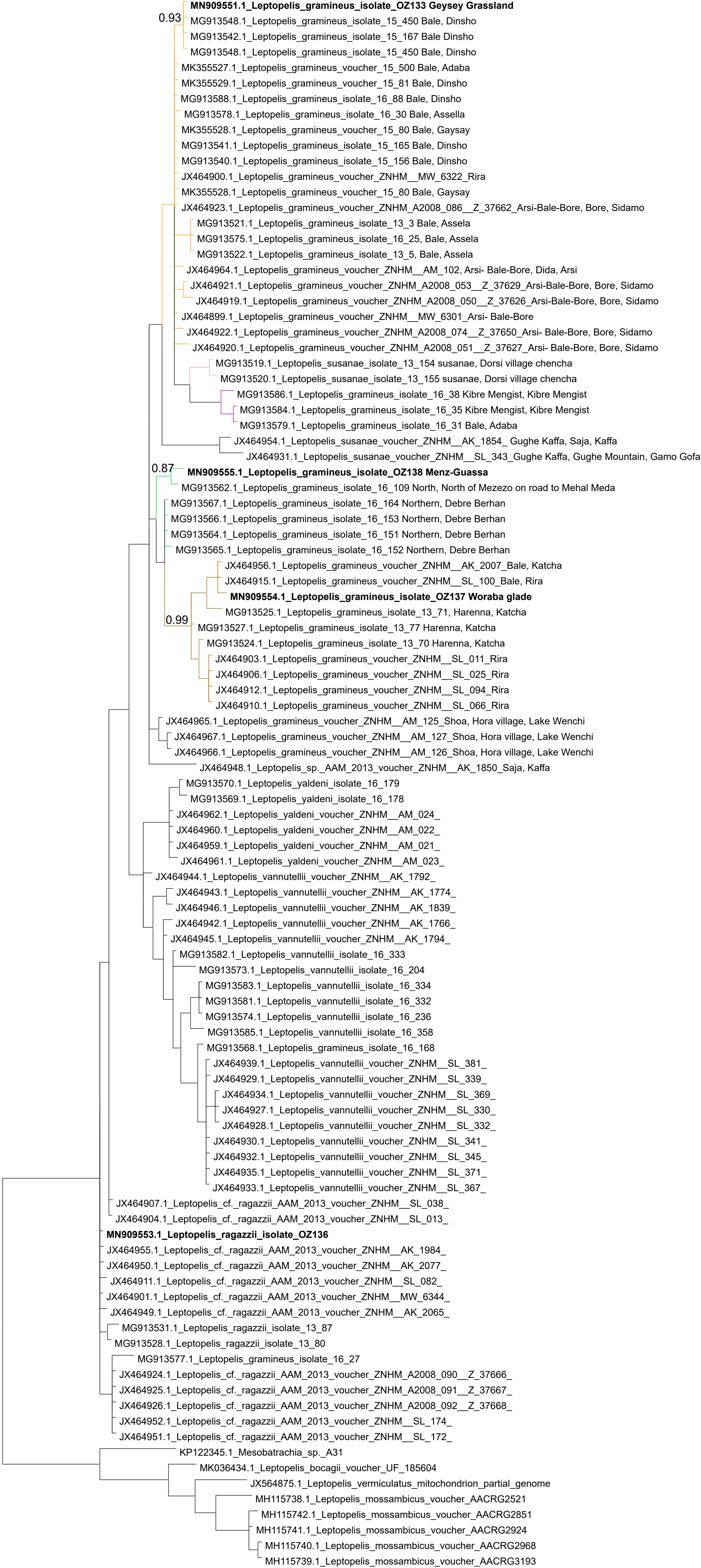

Supplement: Supplementary material 11 — Phylogenetic tree [file zookeys-1023-119-s011.pdf]

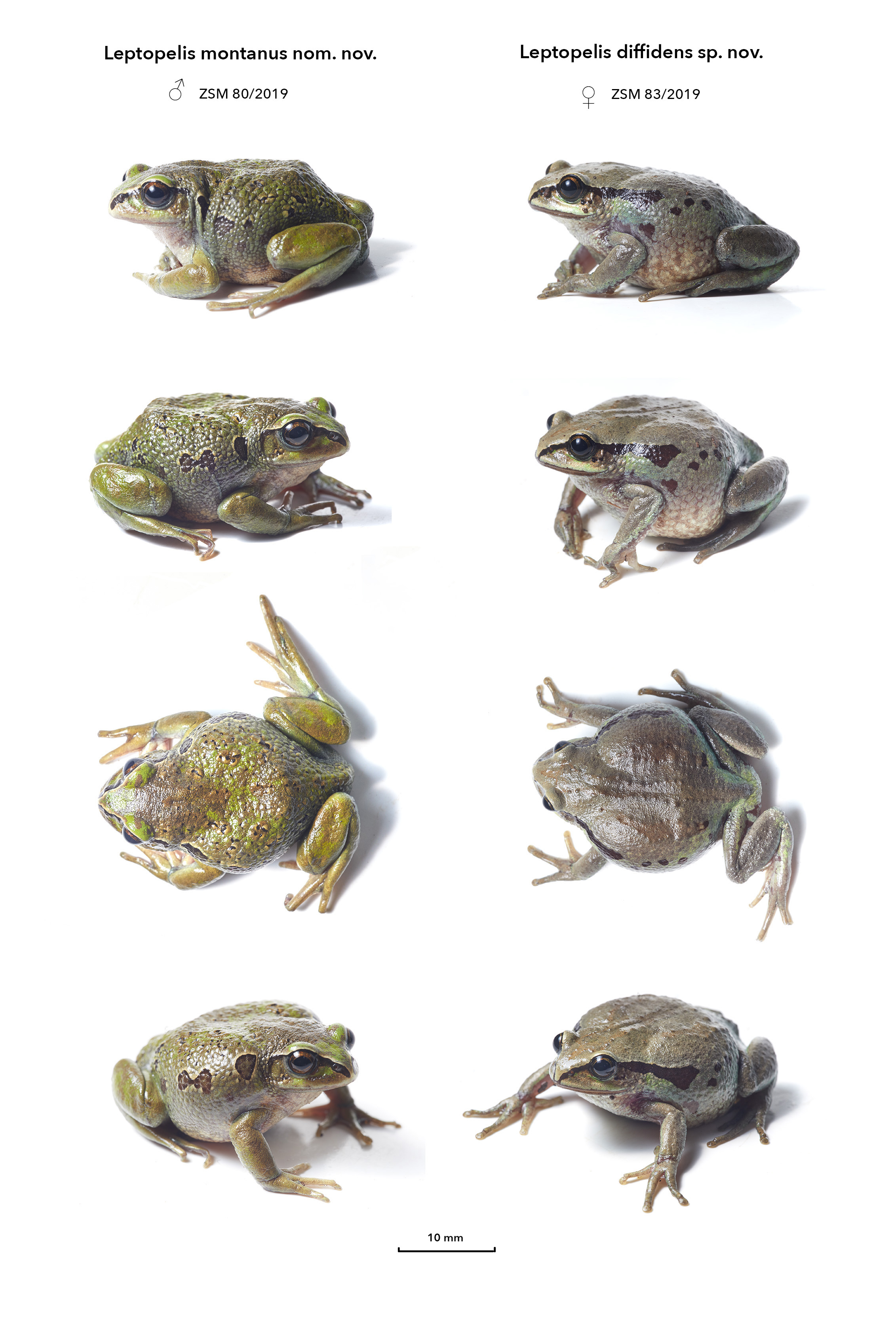

Supplement: Supplementary material 12 — Comparison of mature female Leptopelis diffidens sp. nov. and mature male L. montanus nom. nov. [file zookeys-1023-119-s012.jpg]
