# Supplementary material for: A new species of Leptopelis (Anura, Arthroleptidae) from the south-eastern slope of the Ethiopian Highlands, with notes on the Leptopelis gramineus species complex and the revalidation of a previously synonymised species
Source: Zookeys. 2021 Mar 11;1023:119–50. doi: 10.3897/zookeys.1023.53404 (PMC7973069; doi:10.3897/zookeys.1023.53404)
Supplement: Supplementary material 6 — Table S2. Examined material [file zookeys-1023-119-s006.pdf]

Table 2. Material examined

Accession number	Instituti on	Sex, age	Locality
		Leptopelis diffidens sp. nov.	
81/2019	ZSM	1 ♀	Segoba glade, Harenna Forest, Ethiopia
82/2019	ZSM	1 juvenile	Woraba glade, Harenna Forest, Ethiopia
83/2019	ZSM	1 ♂	Woraba glade, Harenna Forest, Ethiopia
172/2019	ZSM	1 larva	Woraba glade, Harenna Forest, Ethiopia
1986.212.198	LIV	1 ♀	Katcha, Harenna Forest, Ethiopia
1986.212.199-222	LIV	24 juveniles	Swamp near Shawe River, Harenna Forest, Ethiopia
		Leptopelis gramineus	
1947.2.10.19-20	BMNH	1 ♂, 1 ♀	between Badditu and Dime, Ethiopia
1975.16.33-34	BMNH	1 ♀, 1 juvenile	4 km N of Dorse, Gamu Gofa, Ethiopia
1975.1618-1619	BMNH	2 ♂	9 km N of Chencha, Gamu Gofa, Ethiopia
1969.193	BMNH	1 ♀	Bonche Valley, Ethiopia
1969.911	BMNH	1 juvenile	Beletta Forest, Ethiopia
		Leptopelis montanus nom. nov.	
1974.2951-2952	BMNH	2 ♀	Dinsho, Bale Mountains, Ethiopia
1973.2140	BMNH	1 juvenile	25 km W of Dinsho, Bale Mountains, Ethiopia
1973.1241	BMNH	1 juvenile	Little Batu. Bale Mountains, Ethiopia
1973.1242	BMNH	1 juvenile	Arsi Mountains, Ethiopia
1973.1649	BMNH	1 juvenile	7 km SE of Goba, Bale Mountains, Ethiopia
1975.1621-1627	BMNH	5 ♂, 2 ♀	Dinsho, Bale Mountains, Ethiopia
1975.1650	BMNH	1 ♂	12 km E of Goba, Bale Mountains, Ethiopia
80/2019	ZSM	1 ♂	Gaysay Grassland, Bale Mountains, Ethiopia
		Leptopelis sp. 'Shewa'	
1887.1.17.14	BMNH	1	Let Merefia, Ethiopia
1902.12.13.169	BMNH	1	Addis Alem, Ethiopia
1927.7.5.36-38	BMNH	3 ♂	Serpent Lake, Wourambouchi, Ethiopia
1969.967-969	BMNH	3 ♀	Kebena River, Addis Ababa, Ethiopia
1973.2163	BMNH	1 ♀	Lekempti, Ethiopia
1974.2953	BMNH	1 ♀	Addis Ababa, Ethiopia

1975.1626	BMNH	1 ♂	S of Debre Sina, Ethiopia
Leptopelis sp. ‘Borana/Sidamo’			
1969.910	BMNH	1 juvenile	N of Kibre Mengist, Ethiopia
1973.2164-2176	BMNH	12 ♂, 1 ♀	SE of Wando, Ethiopia
1975.1629-1632	BMNH	2 ♂, 2 ♀	23 km SE of Kibre Mengist, Ethiopia
1975.1635-1648	BMNH	11 ♂, 3 ♀	70 km NW of Kibre Mengist, Ethiopia
1975.1669-1670	BMNH	2 ♂	7 km NW of Kibre Mengist, Ethiopia
A-003712	PEM	1 ♂	18 km from Wabi Shabeli, Ethiopia
A-003785-86	PEM	1 ♂, 1 ♀	9 km W of Wabi Shabeli River, Ethiopia
A-003799	PEM	1 ♂	13 km W of Wabi Shabeli River, Ethiopia
A-003800	PEM	2 ♂	20 km W of Wabi Shabeli River bridge, Ethiopia
A-003818-21	PEM	3 ♂, 1 ♀	26 km W of Wabi Shabeli River bridge, Ethiopia
Leptopelis ragazzii			
1986.212.178-181	LIV	12 ♂, 2 ♀	Katcha, Harenna Forest, Ethiopia
284/2018	ZSM	1 ♀	SE of Wajitu Shabi, Harena Forest, Ethiopia
63-64/2019	ZSM	2 ♂	Aboye clearing, Harenna Forest, Ethiopia
Leptopelis susanae			
1976.1023-24	BMNH	2 ♂	4 km N of Dorse Gamu Gofa, Ethiopia
1976.1005-1022	BMNH	15 ♂, 2 ♀	9 km N of Chencha, Gamu Gofa, Ethiopia
Leptopelis yaldeni			
1988.168	LIV	1 ♂	Debre Marcos, Ethiopia
Leptopelis vannutellii			
1974.2997-2999	BMNH	3 ♂	5 km E Scecchi River bridge near Yaiyo, Ethiopia
1974.2983-2996	BMNH	11 ♂, 3 ♀	436 km on Jimma-Bonga road, Keffa, Ethiopia
Leptopelis bocagii			
1976.1111	BMNH	1 juvenile	Godare, Illubabor, Ethiopia
1976.962-966	BMNH	1 ♂, 2 ♀, 2 juveniles	Gambela, Illubabor, Ethiopia
1975.1607-1617	BMNH	9 ♂, 2 ♀	28 km N of Sidamo-Bale Bridge, Bale, Ethiopia
1969.1135-1149	BMNH	18 juveniles	Bahir Dar, Amhara, Ethiopia
Leptopelis concolor			
1994.787	BMNH	1 ♂	Litipo Forest Reserve, Lindi district, Lindi Region, Tanzania
1936.10.1.4-5	BMNH	1 ♂, 1 ♀	Near Witu, Kenya
