# Supplementary material for: A new species of Leptopelis (Anura, Arthroleptidae) from the south-eastern slope of the Ethiopian Highlands, with notes on the Leptopelis gramineus species complex and the revalidation of a previously synonymised species
Source: Zookeys. 2021 Mar 11;1023:119–50. doi: 10.3897/zookeys.1023.53404 (PMC7973069; doi:10.3897/zookeys.1023.53404)
Supplement: Supplementary material 7 — Table S3. Gazetteer [file zookeys-1023-119-s007.pdf]

Toponym	Longitude (N)	Latitude (E)	Elevation (m)
Abaya, Lake	06°26'	37°53'	1175
Aboye	6°41'21"	39°43'31"	2050
Arba Minch	06°02'	37°33'	1285
Assela	7°57’10"	39°07'53"	2500
Bahir Dar	11°33'	37°22'	1800
Badditu	05°50′	37°50′	
Berbere	06°40'	40°10'	
Bonche Valley	06°06'	37°22'	3000
Bonga	07°16′	36°14′	1710
Bore	06°15'	38°45'	1800-2900
Burji	05°30′	37°50′	
Chamo, Lake	05°50'	37°33'	1110
Chencha	06°15'	37°34'	2730
Chencha, 9 km N of	06°20'	37°40'	2700
Debre Berhan	9°40'43"	39°31'59"	2830
Debre Marcos	10°20'	37°44'	2445
Debre Sina, S of	09°50'	39°44'	3300
Dida Plateau	07°11'	39°15'	
Dime	06°16'	36°22'	
Dinsho	7°05'39"	39°47'28"	3100
Dorse	06°16'	37°33'	2829
Dorse, 4 km N of	06°14'	37°38'	2800
Fiche	9°47’12"	38°43'58"	2738
Gambela	08°15'	34°35'	515
Gaysay Grasslands	7°06'56"	39°44'58"	2990
Gecha	6°34'45"	35°27'19"	
Goba, 12 km E of	06°51'	40°03	3900
Godare	07°26'	35°00'	820
Gamo Gofa	06°36'	37°09'	
Gola	06°41'06"	39°38'58"	1850
Gughe, Mountain	06°12'	37°30'	4200
Hacho	06°41'12"	39°39'47"	1900
Harawa	06°38'52"	39°36'55"	1750
Haro Alati	06°26'08"	39°45'59"	1430
Hordoba	06°41'21"	39°39'09"	1890
Kaffa Guasaa	06°41'20"	39°39'20"	1880
Katcha	06°43'01"	39°43'33"	2410
Kibre Mengist	05°52'38"	38°59'20"	1750
Kibre Mengist, 70 km NW of	06°23'	38°35'	2650
Kibre Mengist, 23 km SE of	05°48'	39°12'	1900
Kofele	07°03'12"	38°47'09"	2690
Koma	7.3180	36.0782	1956
Ladamo Mountain	06°38'14"	39°30'36"	3220

Toponym	Longitude (N)	Latitude (E)	Elevation (m)
Lekempti	09°04'	36°33'	2150
Let Marefia	09°39'	39°46'	3000
Mankira	7.1956	36.2844	2647
Manyate	06°29'07"	39°44'44"	1530
Megano	6°38'20"	39°44'02"	1900
Menz-Guassa	10°17'41"	39°47'59"	3250
Meta	09°10'	38°15'	2500
Mezezo, N of	10°09’53"	39°45’53”	3167
Mizan Teferi	7°00’21”	35°42'43"	2300
Rira	06°45'28"	39°43'23"	3000
Saja	7°30’06"	36°05'30"	1965
Sanetti Plateau	06°47'	39°49'	~4100
Segoba	06°35'10"	39°44'30"	1770
Shawe River	6°38'45"	39°43'55"	1940
Sire	06°33'31"	39°42'18"	1670
Smith, Mountain	06°13'21"	36°20'18"	2530
Wando	06°36'	28°25'	1930
Wajitu Shabi, SE of	06°55'05"	40°07'47"	2630
Wabi Shabeli river, 18 km from	07°06'	38°55'	
Wabi Shabeli river, 9 km W of	06°45'	38°48'	
Web Valley	07°00'51"	39°43'27"E	3440
Wondo Genet	7.0987	38.6404	1966
Wonchi, Lake	08°50′34″	38°0′40″	3450
Woraba	06°35'37"	39°45'11"	1800
Wouramboulchi	09°02'	37°50'	2800
Yagana	06°38'55"	39°37'23"	1800
