# Supplementary material for: A new species of Leptopelis (Anura, Arthroleptidae) from the south-eastern slope of the Ethiopian Highlands, with notes on the Leptopelis gramineus species complex and the revalidation of a previously synonymised species
Source: Zookeys. 2021 Mar 11;1023:119–50. doi: 10.3897/zookeys.1023.53404 (PMC7973069; doi:10.3897/zookeys.1023.53404)
Supplement: Supplementary material 10 — Principal component analysis plot [file zookeys-1023-119-s010.pdf]

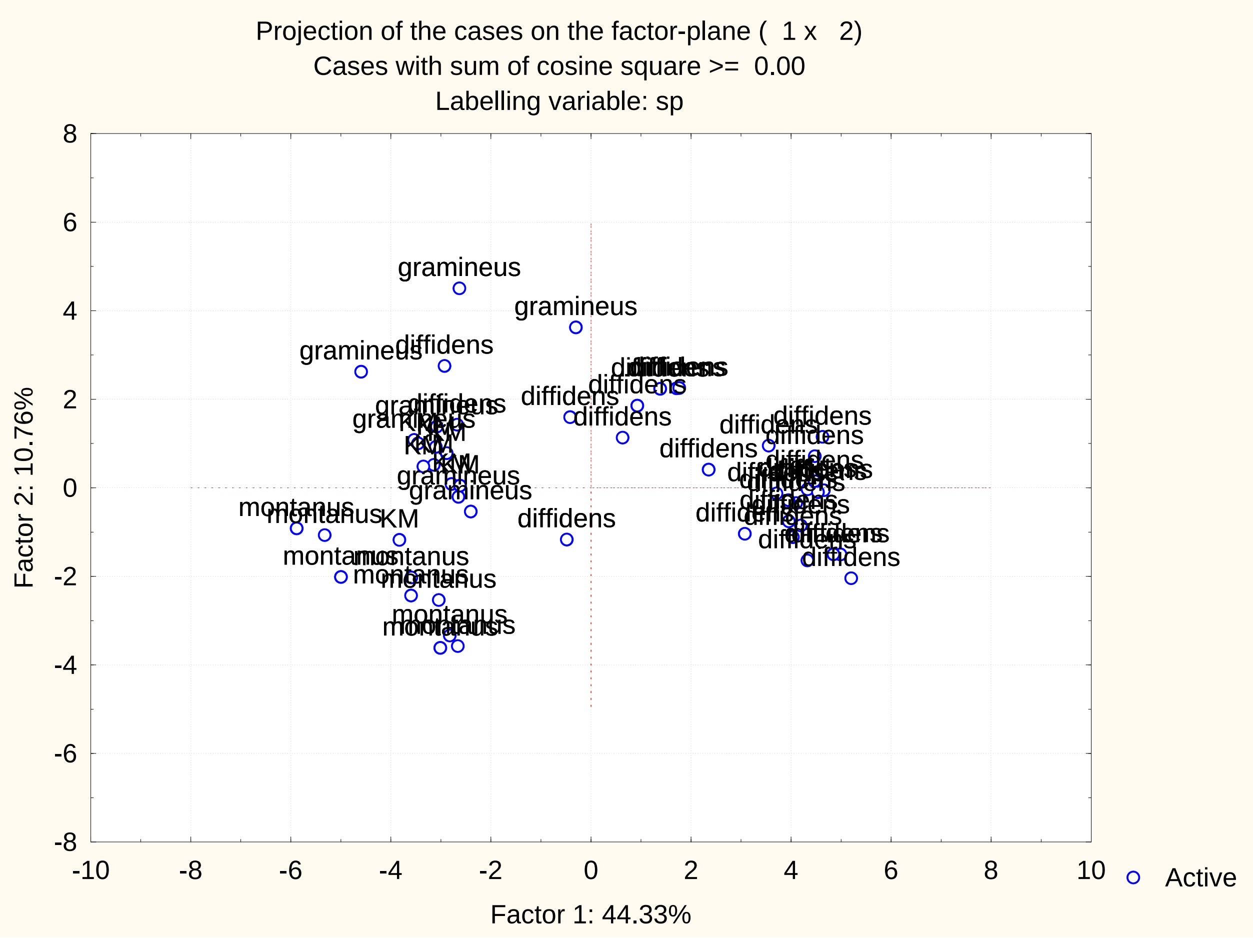
Projection of the cases on the factor-plane (1 x 2)
Cases with sum of cosine square >= 0.00
Labelling variable: sp
Factor 1: 44.33%
Factor 2: 10.76%
-10
-8
-6
-4
-2
0
2
4
6
8
10
Active
gramineus
diffidens
KM
montanus
